# Abscisic acid–induced transcription factor PsMYB306 negatively regulates tree peony bud dormancy release

**DOI:** 10.1093/plphys/kiae014

**Published:** 2024-01-11

**Authors:** Yanping Yuan, Lingling Zeng, Derong Kong, Yanxiang Mao, Yingru Xu, Meiling Wang, Yike Zhao, Cai-Zhong Jiang, Yanlong Zhang, Daoyang Sun

**Affiliations:** College of Landscape Architecture and Arts, Northwest A&F University, Yangling, Shaanxi 712100, China; College of Landscape Architecture and Arts, Northwest A&F University, Yangling, Shaanxi 712100, China; College of Landscape Architecture and Arts, Northwest A&F University, Yangling, Shaanxi 712100, China; College of Landscape Architecture and Arts, Northwest A&F University, Yangling, Shaanxi 712100, China; College of Landscape Architecture and Arts, Northwest A&F University, Yangling, Shaanxi 712100, China; College of Landscape Architecture and Arts, Northwest A&F University, Yangling, Shaanxi 712100, China; College of Landscape Architecture and Arts, Northwest A&F University, Yangling, Shaanxi 712100, China; Department of Plant Sciences, University of California, Davis, Davis, CA 95616, USA; Crops Pathology and Genetics Research Unit, USDA-ARS, Davis, CA 95616, USA; College of Landscape Architecture and Arts, Northwest A&F University, Yangling, Shaanxi 712100, China; College of Landscape Architecture and Arts, Northwest A&F University, Yangling, Shaanxi 712100, China

## Abstract

Bud dormancy is a crucial strategy for perennial plants to withstand adverse winter conditions. However, the regulatory mechanism of bud dormancy in tree peony (Paeonia suffruticosa) remains largely unknown. Here, we observed dramatically reduced and increased accumulation of abscisic acid (ABA) and bioactive gibberellins (GAs) GA_1_ and GA_3_, respectively, during bud endodormancy release of tree peony under prolonged chilling treatment. An Illumina RNA sequencing study was performed to identify potential genes involved in the bud endodormancy regulation in tree peony. Correlation matrix, principal component, and interaction network analyses identified a downregulated MYB transcription factor gene, *PsMYB306*, the expression of which positively correlated with *9-CIS-EPOXYCAROTENOID DIOXYGENASE 3* (*PsNCED3*) expression. Protein modeling analysis revealed 4 residues within the R2R3 domain of PsMYB306 to possess DNA binding capability. Transcription of *PsMYB306* was increased by ABA treatment. Overexpression of *PsMYB306* in petunia (Petunia hybrida) inhibited seed germination and plant growth, concomitant with elevated ABA and decreased GA contents. Silencing of *PsMYB306* accelerated cold-triggered tree peony bud burst and influenced the production of ABA and GAs and the expression of their biosynthetic genes. ABA application reduced bud dormancy release and transcription of *ENT-KAURENOIC ACID OXIDASE 1* (*PsKAO1*), *GA20-OXIDASE 1* (*PsGA20ox1*), and *GA3-OXIDASE 1* (*PsGA3ox1*) associated with GA biosynthesis in *PsMYB306*-silenced buds. In vivo and in vitro binding assays confirmed that PsMYB306 specifically transactivated the promoter of *PsNCED3*. Silencing of *PsNCED3* also promoted bud break and growth. Altogether, our findings suggest that PsMYB306 negatively modulates cold-induced bud endodormancy release by regulating ABA production.

## Teaser

A MYB transcription factor inhibits tree peony bud dormancy release by modulating abscisic acid production.

## Introduction

During the annual growth cycle of perennial plants, bud dormancy can be considered as the inability of the meristems to resume growth under favorable conditions. It is an effective strategy to adapt to the winter environments, such as cold and drought, for the plant survival under harsh conditions (Rohde and Bhalerao 2007; Cooke et al. 2012). Bud dormancy can be classified into the endo-, para-, and ecodormancy (Gillespie and Volaire 2017). Of them, the state of bud formation and meristem quiescence is called endodormancy. Low temperature and short-day length are 2 main signals that induce endodormancy and growth arrest (Yang et al. 2021). In many temperature-sensitive plants, endodormant buds can recover their growth potential after exposure to low temperature for a certain period of time (Anderson et al. 2010). Thus, the release of endodormancy is the prerequisite for the bud break under favorable climatic conditions. It is also a crucial developmental step that affects bud sprouting, plant growth, and flowering (Yamane et al. 2023).

It is well recognized that bud endodormancy is regulated by various phytohormone signals, especially abscisic acid (ABA) and gibberellins (Gas; Yang et al. 2019). It has been known that ABA content varies during endodormancy establishment, maintenance, and release by modifying a series of metabolic processes. For example, ABA levels increased at the initial stage of dormancy and then gradually decreased as the dormancy was relieved in grapevine (*Vitis vinifera*) buds (Zheng et al. 2015). The corresponding expression patterns of ABA biosynthetic and signaling genes have been identified in different woody species, such as tea (*Camellia sinensis*; Yue et al. 2018), peach (*Prunus persica*; Wang et al. 2016), and pear (*Pyrus pyrifolia*; Tuan et al. 2017). In pear, the application of exogenous ABA promoted growth cessation and accelerated the transition to dormancy (Li et al. 2018). The treatment with ABA on apple (*Malus domestica*) and grapevine plants has been shown to be effective for inducing deeper dormancy and facilitating the occurrence of dormancy-associated physiological events (Guak and Fuchigami 2001; Li and Dami 2016). GAs also play a critical role in the modulation of bud dormancy, as a substantial change in bioactive GA levels during dormancy release has been reported (Rohde and Bhalerao 2007). In general, endogenous GA levels are reduced during endodormancy induction and increased during endodormancy release. Changes in GA contents have been reported in hybrid aspen (*Populus tremula* × *Populus tremuloides*; Rinne et al. 2011), grapevine (Zheng et al. 2018), and Japanese apricot (*Prunus mume*; Zhuang et al. 2013). In Populus, some GA biosynthetic genes were upregulated during bud burst, and bioactive GAs GA_3_ and GA_4_ showed different efficiencies for inducing endodormancy release (Rinne et al. 2011). However, GAs inhibited bud endodormancy release and promoted bud primordia growth after dormancy release in grapevine, suggesting a complex role of GAs in the dormancy regulation (Zheng et al. 2018). It has been demonstrated that the endodormancy was maintained by ABA-mediated repression of bud meristem activation, and the removal of this repression triggered the endodormancy release in grapevine buds (Zheng et al. 2018). A previous report has revealed that GA_3_ had the most obvious effect on bud endodormancy release in tree peony (Paeonia suffruticosa), indicating that GA_3_ may be the key bioactive GA in promoting bud break (Zhang, Yuan, et al. 2021). Although the crosstalk between ABA and GAs is well known (Veerabagu et al. 2023), their roles in the regulation of bud endodormancy have not been thoroughly elucidated.

In addition, a massive transcriptional reprogramming occurs during developmental or forced bud burst (Pacey-Miller et al. 2003; Halaly et al. 2008; Yang et al. 2019). In hybrid aspen, the cold-responsive SHORT VEGETATIVE PHASE-LIKE (SVL) and its downstream target TEOSINTE BRANCHED1/CYCLOIDEA/PCF 18 (TCP18) functioned as the key components of bud break that antagonistically acts on the GA and ABA pathways (Singh et al. 2018). In pear, a GA-stimulated transcript (GAST), PpyGAST1, was implicated in the GA biosynthesis to modulate bud dormancy release (Yang et al. 2019). PpDAM1, a dormancy-associated MADS-box (DAM) protein, specifically upregulated the expression of 9-CIS-EPOXYCAROTENOID DIOXYGENASE 3 (PpNCED3) and participated in the ABA-mediated dormancy regulation (Bai et al. 2013).

MYB proteins constitute the largest transcription factor (TF) family in plants. They are well known for the important roles in the accumulation of anthocyanins in plants (Naing and Kim 2018). However, there are few reports on MYB TFs regulating the bud dormancy in perennial plants. In sweet cherry (Prunus avium), a number of PavMYB genes were highly expressed in the dormancy-inducing phase of fruits and flowers (Sabir et al. 2022). Previous studies have demonstrated that 3 MYB genes showed higher expression levels during dormancy transition in tea plants (Hao et al. 2017). PpMYB52 negatively regulated the process of peach bud break in response to GAs (Zhang et al. 2022). To date, the detailed regulatory mechanism of bud dormancy by MYB TFs is still unknown.

Tree peony (Paeonia section Moutan DC.) is one of the most famous traditional flowers originating in China with high ornamental values (Guo et al. 2020). Like many perennial plants, endodormant tree peony must undergo a prolonged chilling or freezing period in winter before bud sprouting (Huang, Xue, et al. 2008). Forcing culture in winter is an important part of tree peony industry to extend the supply of floral products. Bud dormancy is the major constraining factor for the achievement of forcing culture. Therefore, the elucidation of molecular basis of bud dormancy is critical for tree peony. In the past decade, several studies have found that bud dormancy release depends on nutrient substance, membrane lipid peroxidation, and dynamic changes of endogenous hormones during the chilling requirement fulfillment. GAs might be the primary signal to initiate the bud dormancy break in tree peony (Gai et al. 2013). As with other species, when tree peony endured insufficient chilling accumulation, exogenous GA_3_ and GA_4_ treatments accelerated bud dormancy release and subsequent growth (Gai et al. 2013). PsMYB1 was reported to be downregulated at the later stage of chilling treatment and was hypothesized to negatively regulate bud dormancy release of tree peony (Zhang et al. 2015). A recent report showed that RGA-LIKE 1 (PsRGL1) encoding a DELLA protein was downregulated during chilling- and GA_3_-induced dormancy release. PsRGL1 participated in the bud dormancy regulation by suppressing GA signaling (Gao et al. 2023). Despite these reports, our understanding of signaling pathways regulating the levels of hormones known to impact bud dormancy in tree peony remains largely unclear.

In previous studies, we performed metabolite and transcriptome analyses during bud sprouting of tree peony under low-temperature stress. Several MYB genes were shown to confer tolerance to freezing by regulating anthocyanin and polyunsaturated fatty acid biosynthesis (Mao et al. 2022). Whether MYB TFs are involved in the regulation of bud dormancy requires a further investigation. Considering the recalcitrance of tree peony plants to genetic transformation, we have successfully used a tobacco rattle virus (TRV)–based virus-induced gene silencing (VIGS) method to investigate the function of genes in tree peony (Xie et al. 2019; Mao et al. 2022). Moreover, petunia (Petunia hybrida) has been widely employed as a model platform for gene overexpression assays in floral crops (Sun et al. 2019, 2020). In this study, we carried out a high-throughput Illumina RNA sequencing (RNA-Seq) to dissect the molecular mechanism of bud endodormancy regulation in tree peony. *PsMYB306*, a member of R2R3-MYB family, was selected from RNA-Seq data for further functional characterization. Our data presented here provided valuable insights into the genetic basis of bud dormancy and would benefit future improvement of forcing culture in tree peony by breeding or bioengineering approach.

## Results

### RNA-Seq reveals the crucial roles of ABA and GAs in chilling-induced bud dormancy release

**Figure 1.**
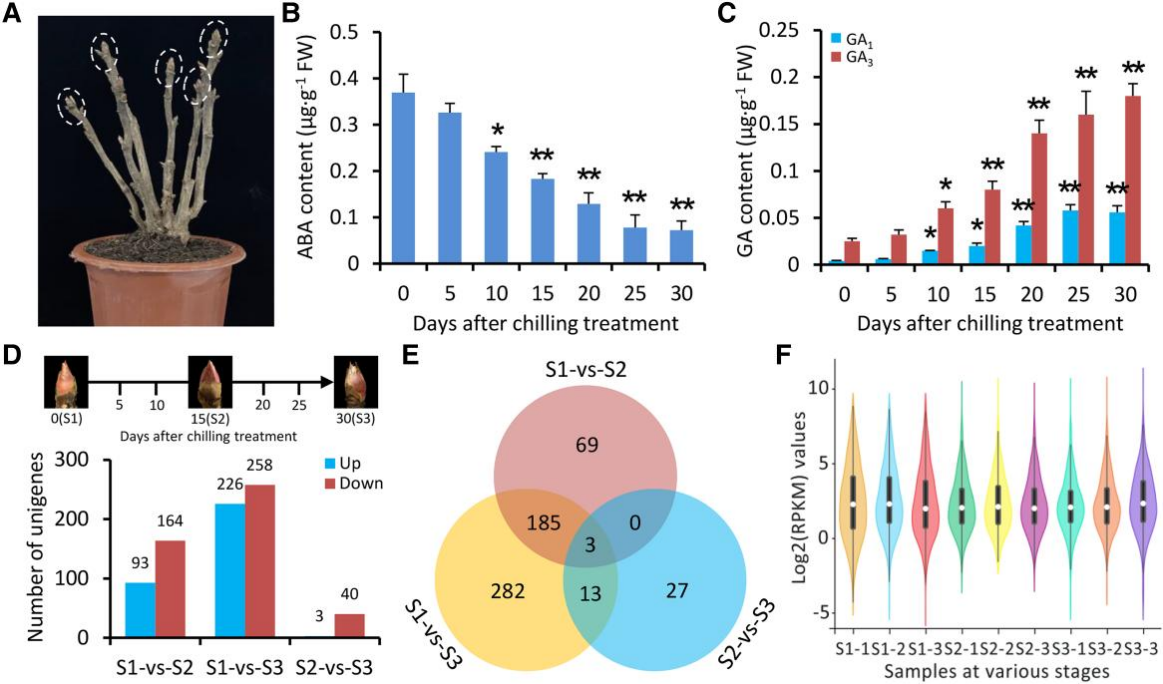
Assessment of RNA-Seq data in tree peony buds treated with chilling. **A)** Representative phenotypes of 5-yr-old grafted tree peony plants used for chilling treatment for promoting the bud endodormancy release. The apical buds are marked in dashed circles. Contents of ABA **B)** and bioactive GAs GA_1_ and GA_3_**C)** in tree peony buds at various days after chilling treatment. **D)** Number of up- and downregulated transcripts in tree peony buds with the pairwise comparisons of S1-vs-S2, S1-vs-S3, and S2-vs-S3. The S1, S2, and S3 stages represent 0, 15, and 30 d after chilling treatment, respectively. **E)** Venn diagram of DEGs for different pairwise comparisons. The shared genes are present in the overlapped regions. **F)** Violin plot of transcript abundances of DEGs in chilling-treated buds, with the abundances being shown as log_2_-transformed RPKM. Hollow circles indicate median values, solid boxes range from the 25th to 75th percentiles, and thin lines connect the lower adjacent value to upper adjacent value. Error bars indicate the Se of the means from 3 biological replicates. Statistical significance was evaluated using Student's *t* test (*P < 0.05, **P < 0.01) and marked by asterisks.

To determine the involvement of endogenous hormones in bud dormancy regulation, the contents of ABA and GAs in tree peony buds (Fig. 1A) upon exposure to chilling stress were quantified. A continuous reduction in ABA accumulation was observed in chilling-treated buds, whereas an elevation in bioactive GA_1_ and GA_3_ levels was found (Fig. 1, B and C). Based on the variation in the hormone levels, 0 (S1), 15 (S2), and 30 (S3) d were considered 3 critical time nodes during chilling-induced bud endodormancy release. To elucidate the regulatory mechanism of bud endodormancy associated with ABA and GAs, we thus performed an Illumina RNA-Seq using the buds from S1 to S3. The sequencing work totally generated 79,300 unigenes (Supplementary Tables S1 and S2). Of them, 35,708, 32,011, 19,291, and 20,993 unigenes were annotated against Nr, Kyoto Encyclopedia of Genes and Genomes (KEGG), COG, and Swiss-Prot databases, respectively (Supplementary Fig. S1A). Principal component analysis (PCA) showed that 2 factors explained 97.1% of the total variance, suggesting substantial differences among samples (Supplementary Fig. S1B).

RNA-Seq analysis revealed 579 differentially expressed genes (DEGs) during chilling-induced bud endodormancy release (Supplementary Table S3). The highest transcriptional divergence was found between S1 and S3, with 226 unigenes upregulated and 258 downregulated. In contrast, the buds exhibited the fewest up- and downregulated unigenes at S3 compared with S2 (Fig. 1D). The pairwise comparisons of S2/S1 and S3/S1 shared a relatively larger number of DEGs (Fig. 1E). Compared with S1 and S3, violin plots showed a different abundance distribution of DEGs on a Log2(RPKM) scale at S2 (Fig. 1F). These findings indicate that transcriptional alteration during bud endodormancy release occurs mainly in the first 2 stages.

**Figure 2.**
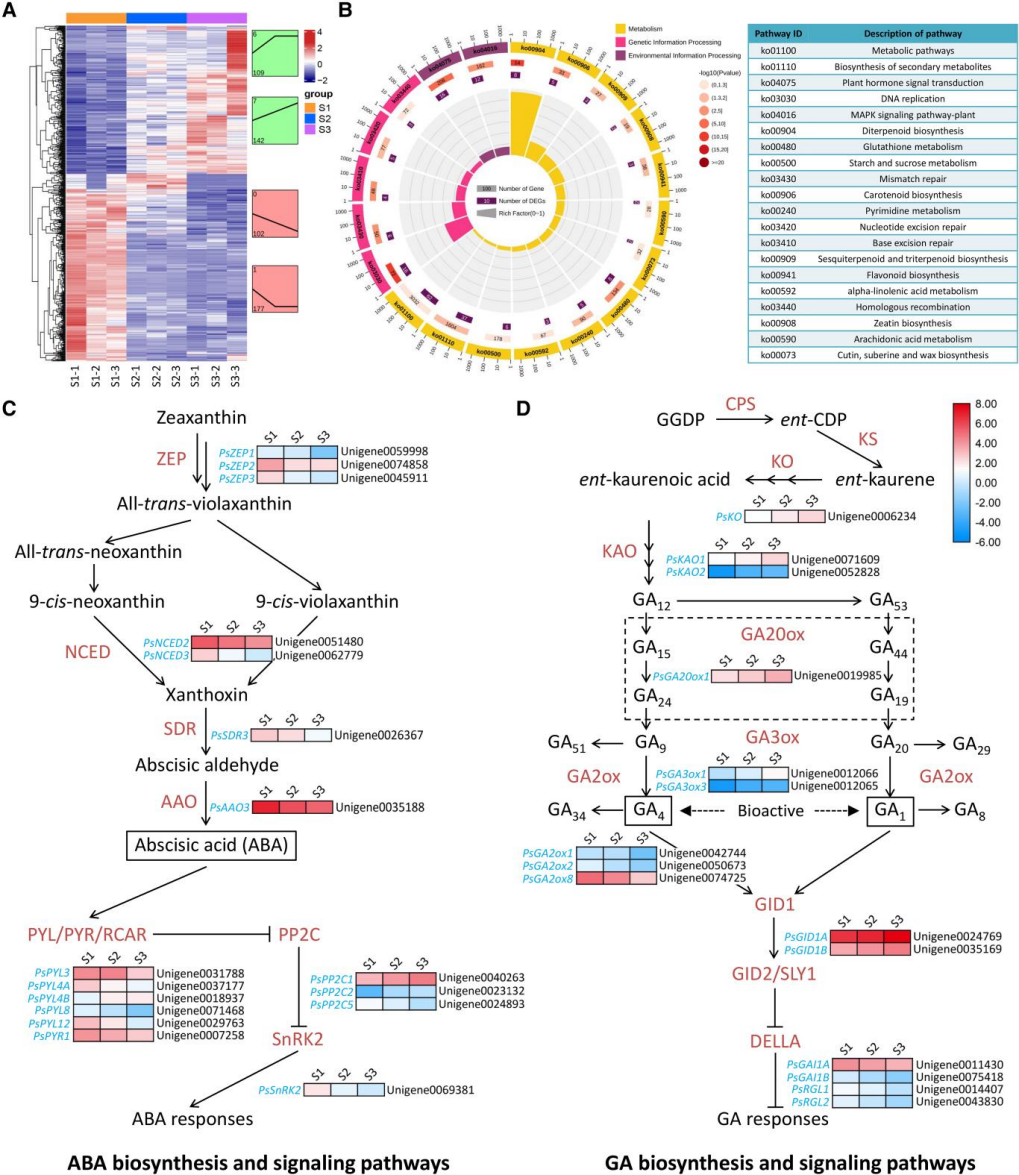
Putative transcripts differentially expressed in chilling-treated tree peony buds through RNA-Seq analysis. **A)** Heatmap of normalized RPKM scaled by the *Z*-score method for all the DEGs. Different colors in the heatmap represent variable RPKMs ranged from low to high levels for each gene. Several primary expression trends of DEGs are shown on the right of heatmap. The profile and DEG numbers are displayed at the top and bottom of each trend graph, respectively. **B)** Enriched top 20 KEGG pathways of DEGs in tree peony buds at 3 stages of chilling treatment. Expression patterns of DEGs in the ABA **C)** and GA **D)** biosynthesis and signaling pathways at 3 stages of chilling-treated tree peony buds. These DEGs are denoted by italics. Different colors in the heatmap represent variable expression ranged from low to high levels for each gene. Solid lines ending with arrows indicate positive regulation, whereas the ones ending with short perpendicular lines represent negative regulation.

Cluster analysis revealed 4 primary profiles for the expression trends of DEGs from S1 to S3. The top 2 profiles (1 and 7) displayed 177 downregulated and 142 upregulated unigenes, respectively (Fig. 2A). KEGG pathway annotation showed that a large number of DEGs were mapped to the pathways of metabolic, biosynthesis of secondary metabolites, and plant hormone signal transduction (Fig. 2B; Supplementary Table S4). Considering the variable accumulation of ABA and GAs in tree peony buds under low-temperature stress, the DEGs in their biosynthesis and signaling pathways were analyzed. Transcript levels of ABA biosynthetic genes *ZEAXANTHIN EPOXIDASE 1–3* (*PsZEP1–3*), *PsNCED2–3*, *SHORT-CHAIN DEHYDROGENASE/REDUCTASE 3* (*PsSDR3*), and *ABA ALDEHYDE OXIDASE 3* (*PsAAO3*) decreased from S1 to S3 under chilling stress. A set of DEGs associated with ABA signaling displayed different expression patterns. Specifically, transcript levels of *PYRABACTIN RESISTANCE-LIKE 3* (*PsPYL3*), *PsPYL4A–B*, *PsPYL8*, *PsPYL12*, and *PYRABACTIN RESISTANCE 1* (*PsPYR1*), which are ABA receptors from the PYL/PYR/RCAP family, and the downstream effector of ABA action *SNF1-RELATED KINASE 2* (*PsSnRK2*) decreased. *PROTEIN PHOSPHATASE 2C 1–2* (*PsPP2C1–2*) and *PsPP2C5*, the negative regulators of ABA signaling, were upregulated (Fig. 2C). For the GA pathways, some GA biosynthetic genes *ENT-KAURENE OXIDASE* (*PsKO*), *ENT-KAURENOIC ACID OXIDASE 1–2* (*PsKAO1–2*), *GA20-OXIDASE 1* (*PsGA20ox1*), *GA3-OXIDASE 1* (*PsGA3ox1*), and *PsGA3ox3* were upregulated, whereas 3 GA catabolic genes *GA2-OXIDASE 1–2* (*PsGA2ox1–2*) and *PsGA2ox8* were downregulated. Correspondingly, a few GA signaling–related genes, including the GA receptors *GA INSENSITIVE DWARF 1A–B* (*PsGID1A–B*) and the DELLA protein-encoding genes *GA INSENSITIVE 1A–B* (*PsGAI1A–B*) and *PsRGL1–2*, showed elevated transcription (Fig. 2D). The expression of DEGs in the ABA and GA pathways was consistent with the production of these hormones.

### *PsMYB306* is downregulated during bud dormancy release and positively correlated with *PsNCED3*

**Figure 3.**
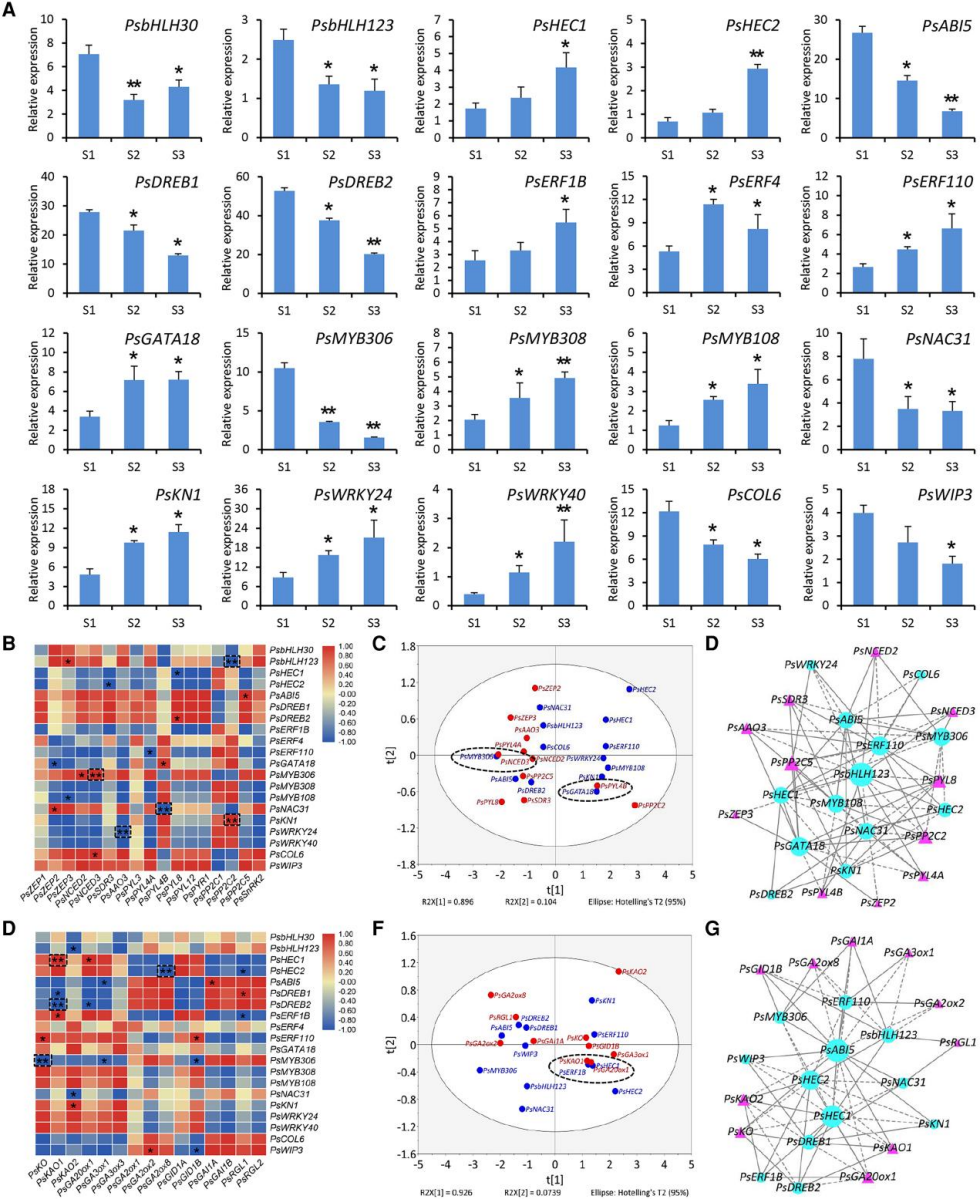
Identification of TFs probably regulating ABA and GA pathway–related genes in chilling-treated tree peony buds. **A)** Transcript levels of 20 differentially expressed TFs at 3 stages of chilling-treated buds based on RNA-Seq data. The correlation matrix–based heatmaps between differentially expressed TFs and the genes in the ABA **B)** and GA **E)** biosynthesis and signaling pathways in chilling-treated buds. Different colors in the heatmap represent variable expression ranged from low to high levels. Dashed squares indicate the correlations at high significance levels. PCA of the selected TFs with the ABA **C)** and GA **F)** biosynthetic and signaling genes in chilling-treated buds. Dashed circles represent the genes with relatively close correlation distance. An interaction network between the selected TFs and ABA **D)** and GA **G)** pathway–associated genes in the buds. Solid or dashed lines represent positive or negative regulation, respectively. Larger circles or triangles with more nodes indicate more significant correlations. Error bars indicate the Se of the means from 3 biological replicates. Significance of difference was determined using Student's *t* test (*P < 0.05, **P < 0.01) and indicated by asterisks.

To understand the transcriptional regulation of dormancy release in tree peony buds, a number of DEGs encoding putative TFs were identified from RNA-Seq data. Functional annotation indicates that they belong to 9 distinct TF families, with most of genes classified into the bHLH, AP2/ERF, and MYB families (Supplementary Table S5). Expression data showed that 11 TFs were upregulated and 9 were downregulated from S1 to S3 under chilling stress. The buds exhibited a more significant change in transcript levels of *PsbHLH30*, *HECATE 2* (*PsHEC2*), *ABA-INSENSITIVE 5* (*PsABI5*), *DRE/CRT-BINDING PROTEIN 2* (*PsDREB2*), *PsMYB306*, *PsMYB308*, and *PsWRKY40* than those of other TFs. In particular, a dramatic 6.7-fold drop in expression levels of *PsMYB306* was observed in the buds from S1 to S3 (Fig. 3A).

Next, Pearson’s correlation analysis between the TFs and DEGs in the ABA or GA biosynthesis and signaling pathways was performed. According to the correlation matrix-based heatmaps, *PsbHLH123*, *PsMYB306*, *PsNAC31*, *KNOTTED 1* (*PsKN1*), and *PsWRKY24* displayed highly significant correlations with a couple of ABA biosynthetic and signaling genes. Of them, *PsMYB306* was found to be positively correlated with *PsNCED3*, a key ABA biosynthetic gene (Fig. 3B). Based on gene expression profiles, the PCA showed that *PsMYB306* was pooled together with *PsNCED3*, revealing a closer transcriptional relationship between them (Fig. 3C). A further interaction network validated a strong positive correlation between *PsMYB306* and *PsNCED3* (Fig. 3D). In terms of the correlations of the TFs with GA pathway-related DEGs, it was found that *PsHEC1*, *PsHEC2*, *PsDREB2*, and *PsMYB306* were correlated at high significance levels with some GA biosynthetic genes, of which *PsKO* was negatively correlated with *PsMYB306* (Fig. 3E). Nevertheless, the PCA and interaction network analysis revealed no significant link between *PsMYB306* and *PsKO* (Fig. 3, F and G). These observations prompt us to hypothesize that PsMYB306 may affect ABA-mediated bud endodormancy release by directly regulating the expression of *PsNCED3*. To verify this hypothesis, PsMYB306 was thus selected for subsequent functional characterization.

### PsMYB306 harbors 4 DNA-binding residues, and its transcription is activated by ABA treatment

**Figure 4.**
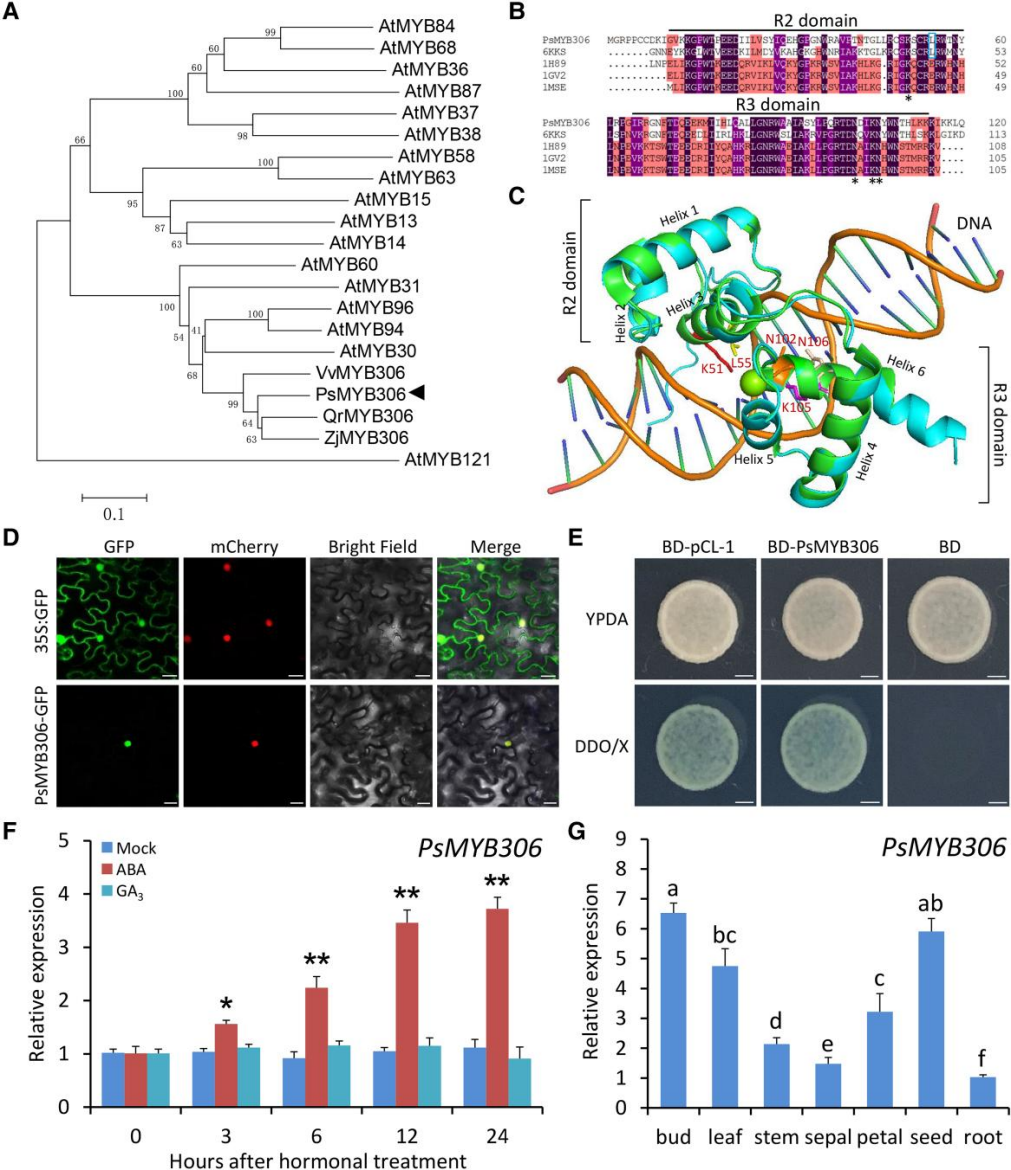
Sequence structure, transcriptional activity, and expression pattern analyses of PsMYB306. **A)** Phylogenetic tree of PsMYB306 with *V. vinifera* VvMYB306, *Q. rubra* QrMYB306, *Z. jujuba* ZjMYB306, and other MYB proteins from Arabidopsis. Bootstrap values are expressed as a percentage of 1,000 replicates and shown at branch nodes. PsMYB306 is marked by a solid triangle. AtMYB121, a member of MYB subgroup 17, served as the outgroup. Scale bar represents 0.1 amino acid substitutions per site. **B)** Amino acid sequence alignment of PsMYB306 with the identified homologous protein templates. Asterisks represent 4 residues related to DNA binding, while the square indicates 1 residue for DNA methylation. **C)** Protein modeling of PsMYB306 in superimposition with Arabidopsis WER (6KKS). The side chains of the corresponding 5 residues in PsMYB306 are shown as sticks. **D)** Subcellular localization of PsMYB306 in *N. benthamiana* leaves based on PsMYB306-GFP fusion. H2B-mCherry was used to mark the nuclei. Scale bars = 20 *μ*m. **E)** Transcriptional activation of PsMYB306 in *S. cerevisiae* cells. The pCL-1 and the empty vector (BD) were used as positive and negative controls, respectively. Scale bars = 15 mm. RT-qPCR analysis of expression levels of *PsMYB306* in the buds treated with 100 *μ*M ABA and 100 *μ*M GA (GA_3_) at intervals **F)** and in different organs or tissues of tree peony **G)**. *PsActin* was used as an internal control. Error bars represent Se of the mean from 3 biological replicates. Asterisks or letters indicate statistical significance as calculated by Student's *t* test (*P < 0.05, **P < 0.01).

Sequence analysis of PsMYB306 revealed that its full-length cDNA contains a complete coding region of 981 bp (Supplementary Fig. S2). PsMYB306 was phylogenetically close to AtMYB96, AtMYB94, and AtMYB30 from Arabidopsis (*Arabidopsis thaliana*) and other MYB306s from grapevine, oak (*Quercus rubra*), and jujube (*Ziziphus jujuba*; Fig. 4A). The typical R2 and R3 domains were shown within the protein sequence of PsMYB306 (Fig. 4B). To determine the critical amino acid residues for PsMYB306's function, we built a high-quality structural model of PsMYB306 using a similar R2R3-MYB protein structure of Arabidopsis WEREWOLF (WER; Protein Data Bank: 6KKS) as the template. A recent report showed that a couple of individual residues in the WER protein are responsible for DNA recognition and methylation (Wang et al. 2020). The spatial positions of these sites were displayed in the generated model. Five residues were distributed in the third helixes of both the R2 and R3 domains. They fitted in the adjacent groove of the DNA, suggesting a putative interaction between 5 residues and target DNA molecules. Through a comparative analysis with WER structure, K51, N102, K105, and N106 in the PsMYB306 protein were predicted to have DNA binding potential, while L55 was pivotal for sensing DNA methylation (Fig. 4C).

Subcellular localization of PsMYB306 was examined through a transient expression of the fusion protein PsMYB306-GFP in *Nicotiana benthamiana* leaves, using H2B-mCherry as a nucleus marker. An overlap between GFP (green) and mCherry (red) fluorescent signals was observed in PsMYB306-expressing cells (Fig. 4D), indicating that PsMYB306 was localized in the nucleus. A transactivation assay was conducted to assess whether PsMYB306 has transcriptional activity. The yeast cells transformed with BD-PsMYB306 grew well and appeared blue on the SD/-Trp-His medium supplemented with X-α-gal. The positive control BD-pCL-1 displayed the same yeast growth and appearance as BD-PsMYB306, whereas the negative control BD failed to grow (Fig. 4E). These findings suggest that PsMYB306 may function as a positive transcriptional regulator. Due to the involvement of ABA and GAs in bud endodormancy regulation, we analyzed the expression pattern of *PsMYB306* in hormone-treated buds. Transcript abundance of *PsMYB306* increased dramatically following ABA treatment but not GA_3_ treatment (Fig. 4F). Besides, *PsMYB306* was expressed in the buds and seeds at higher levels than other tree peony tissues (Fig. 4G).

### Overexpression of *PsMYB306* inhibits seed germination and plant growth in petunia

**Figure 5.**
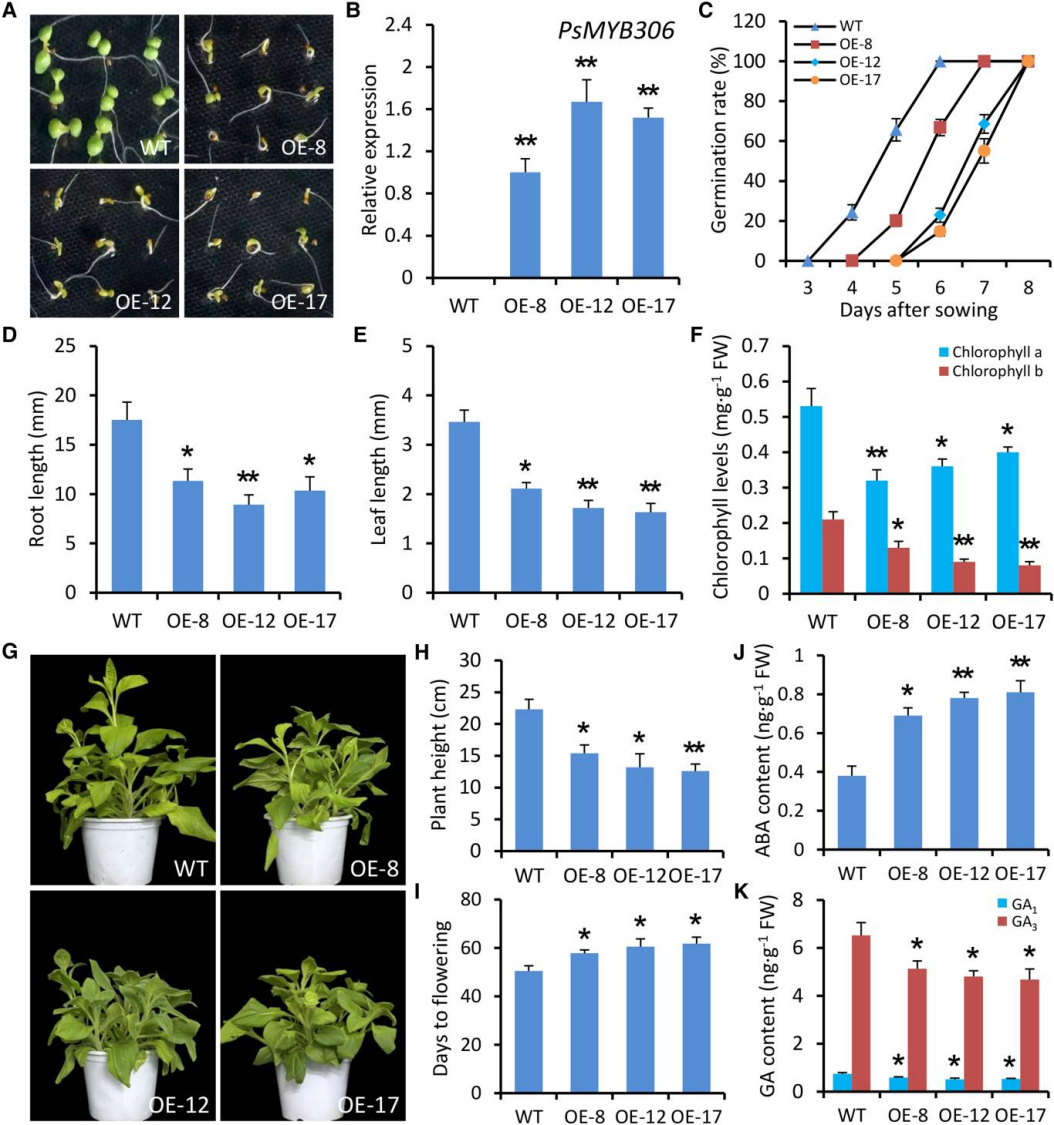
Overexpression of *PsMYB306* inhibits seed germination and plant growth in petunia. **A)** Representative phenotypes of germinating seeds from WT and *PsMYB306*-overexpressing (OE) transgenic petunia plants at 10 DAS. The freshly harvested seeds were surface sterilized with 2% NaClO for 10 min and washed 3 times with sterile water and then transferred onto the 1/2 MS medium. **B)** RT-qPCR analysis of expression levels of *PsMYB306* in young leaves from WT and transgenic petunia lines at 10 DAS. **C)** Germination rates of the seeds from WT and transgenic petunia lines OE *PsMYB306* at different time points. Root lengths **D)**, leaf lengths **E)**, and chlorophyll levels **F)** in WT and transgenic petunia lines at 10 DAS. The leaf tissues were used for chlorophyll measurement. **G)** Representative phenotypes of WT and *PsMYB306*-OE transgenic petunia plants. The plants were digitally extracted for comparison. Growth heights **H)** and flowering time **I)** of transgenic petunia lines in comparison with WT controls. Contents of ABA **J)** and bioactive GAs GA_1_ and GA_3_**K)** in young leaves from WT and transgenic lines. The plants at 50 DAS were used for phenotypic comparison, growth measurement, and hormone examination. Expression levels were standardized to *PhEF1α*. Error bars represent Se of the mean from 3 biological replicates. Asterisks indicate statistical significance as determined by Student's *t* test (*P < 0.05, **P < 0.01).

To investigate the function of PsMYB306 in the dormancy regulation, we carried out a heterologous transformation experiment in petunia. *PsMYB306*-overexpressing transgenic petunia lines exhibited repressed seed germination compared with wild-type (WT) lines (Fig. 5A). A substantial transcription of *PsMYB306* in transgenic petunia plants was confirmed through reverse transcription quantitative PCR (RT-qPCR) analysis (Fig. 5B). The seed germination rates in the overexpressing lines were much lower than those in the WT lines at different days after sowing (DAS; Fig. 5C). *PsMYB306* overexpression also led to decreased lengths of root and leaf and levels of chlorophyll a and b in the leaves (Fig. 5, D to F). These are in accordance with the delayed seed germination and leaf yellowing phenotypes in transgenic lines overexpressing *PsMYB306*. In comparison with WT controls, the growth of petunia plants was affected due to the overexpression of *PsMYB306* (Fig. 5G), leading to remarkably reduced plant heights (Fig. 5H). The duration from sowing to initial flowering was extended in transgenic petunia plants (Fig. 5I). To determine the association of phenotypic variation with hormonal signals, the production of dormancy-related ABA and GAs was examined. As shown in Fig. 5, J and K, *PsMYB306*-overexpressing transgenic lines displayed elevated ABA content and reduced contents of bioactive GAs GA_1_ and GA_3_.

### Silencing of *PsMYB306* promotes bud dormancy release of tree peony

**Figure 6.**
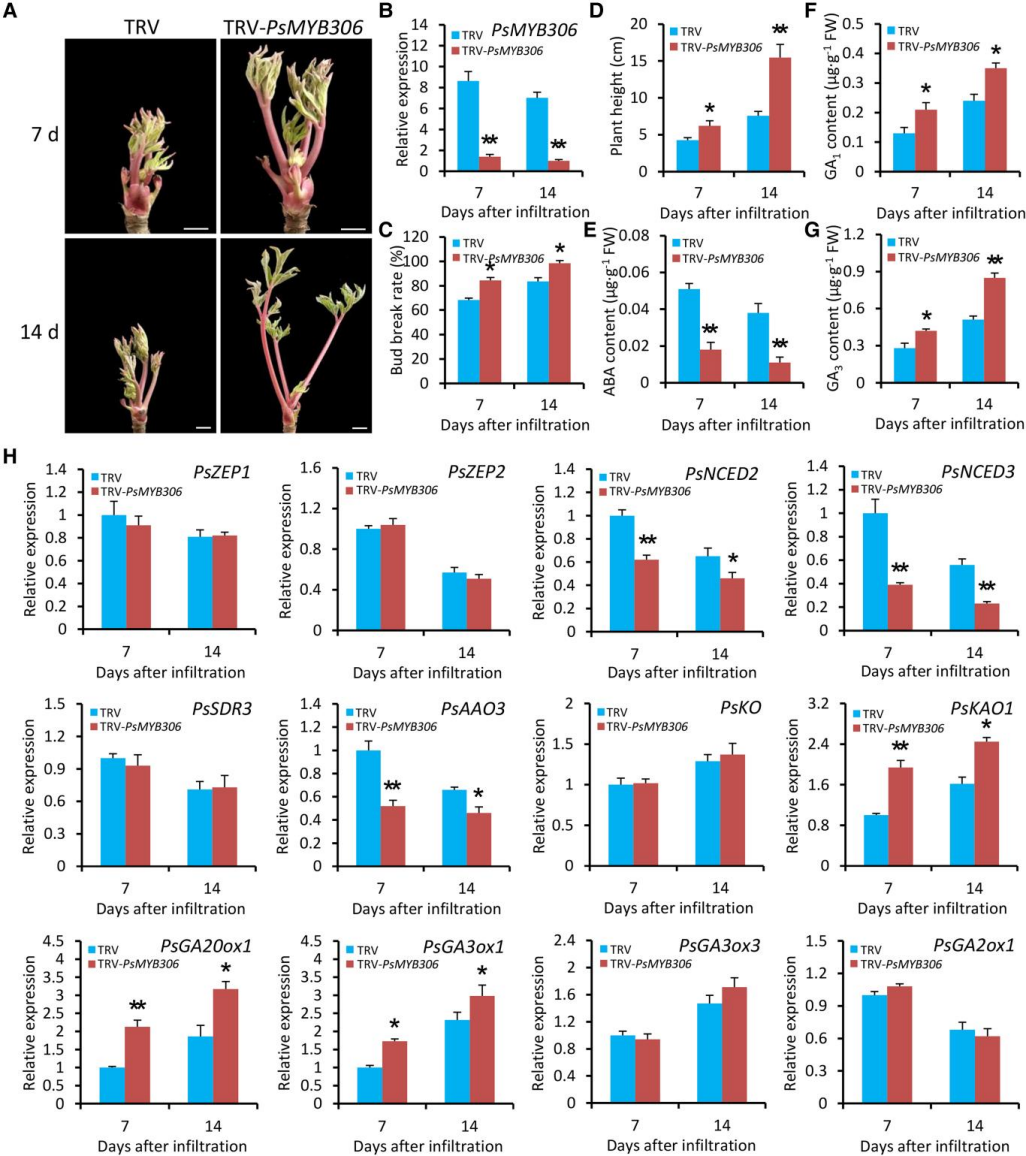
Silencing of *PsMYB306* accelerates bud dormancy release in tree peony. **A)** Representative phenotypes of sprouting buds from 1-yr-old grafted tree peony plants infiltrated with TRV empty vector or TRV-*PsMYB306*. The chilling-treated buds for 25 d were used in the VIGS assay. Photographs were taken at 7 and 14 DAI. The buds were digitally extracted for comparison. Scale bars = 0.8 cm. **B)** RT-qPCR analysis of expression levels of *PsMYB306* in the buds inoculated with various TRV constructs at 7 and 14 DAI. Bud break rates **C)** and growth heights **D)** of TRV empty vector- and TRV-*PsMYB306*-infected tree peony plants at 7 and 14 DAI. Contents of ABA **E)** and bioactive GAs GA_1_ and GA_3_**F, G)** in the buds infected with various TRV constructs at given time points. **H)** RT-qPCR analysis of expression levels of a number of ABA and GA biosynthetic genes in TRV empty vector- and TRV-*PsMYB306*-infected tree peony plants at various time points. Transcript abundances were normalized to *PsActin*. Error bars represent Se of the mean from 3 biological replicates. Statistical significance was verified using Student's *t* test (*P < 0.05, **P < 0.01) and denoted by asterisks.

To further dissect the role of PsMYB306 in the dormancy release, a TRV-based VIGS approach was used to knockdown *PsMYB306* in tree peony buds exposed to cold stress. A sufficient viral RNA accumulation was found in systemically infected leaves through RT-qPCR analysis, supporting the effectiveness of VIGS system in tree peony plants (Supplementary Fig. S3). An early sprouting and accelerated growth occurred in the buds at 7 and 14 d after inoculation with TRV-*PsMYB306* compared with empty vector control (Fig. 6A). RT-qPCR analysis revealed a reduced transcription of *PsMYB306* in TRV-*PsMYB306*-infected buds (Fig. 6B). *PsMYB306*-silenced buds showed significantly higher bud break rates and plant heights than the control ones (Fig. 6, C and D). For the hormonal assessment, the ABA production was decreased in the buds with *PsMYB306* silencing (Fig. 6E), but the accumulation of bioactive GAs GA_1_ and GA_3_ was promoted (Fig. 6, F and G). These results were opposite to those found in *PsMYB306*-overexpressing petunia plants, indicating that PsMYB306 played a crucial role in bud dormancy release. To deeply understand the regulation of ABA and GA pathways by PsMYB306, we examined the transcription of some genes associated with ABA and GA biosynthesis. Silencing of *PsMYB306* caused a reduction in transcript abundances of ABA biosynthetic genes *PsNCED2–3* and *PsAAO3* and an elevated expression of GA biosynthetic genes *PsKAO1*, *PsGA20ox1*, and *PsGA3ox1*. However, no significant change in the transcription of *PsZEP1–2*, *PsSDR3*, *PsKO*, *PsGA3ox3*, and *PsGA2ox1* was observed (Fig. 6H). Thus, we speculated that PsMYB306 probably affected bud endodormancy release by regulating the crosstalk between ABA and GAs.

### Exogenous application with ABA represses GA-mediated bud dormancy release

**Figure 7.**
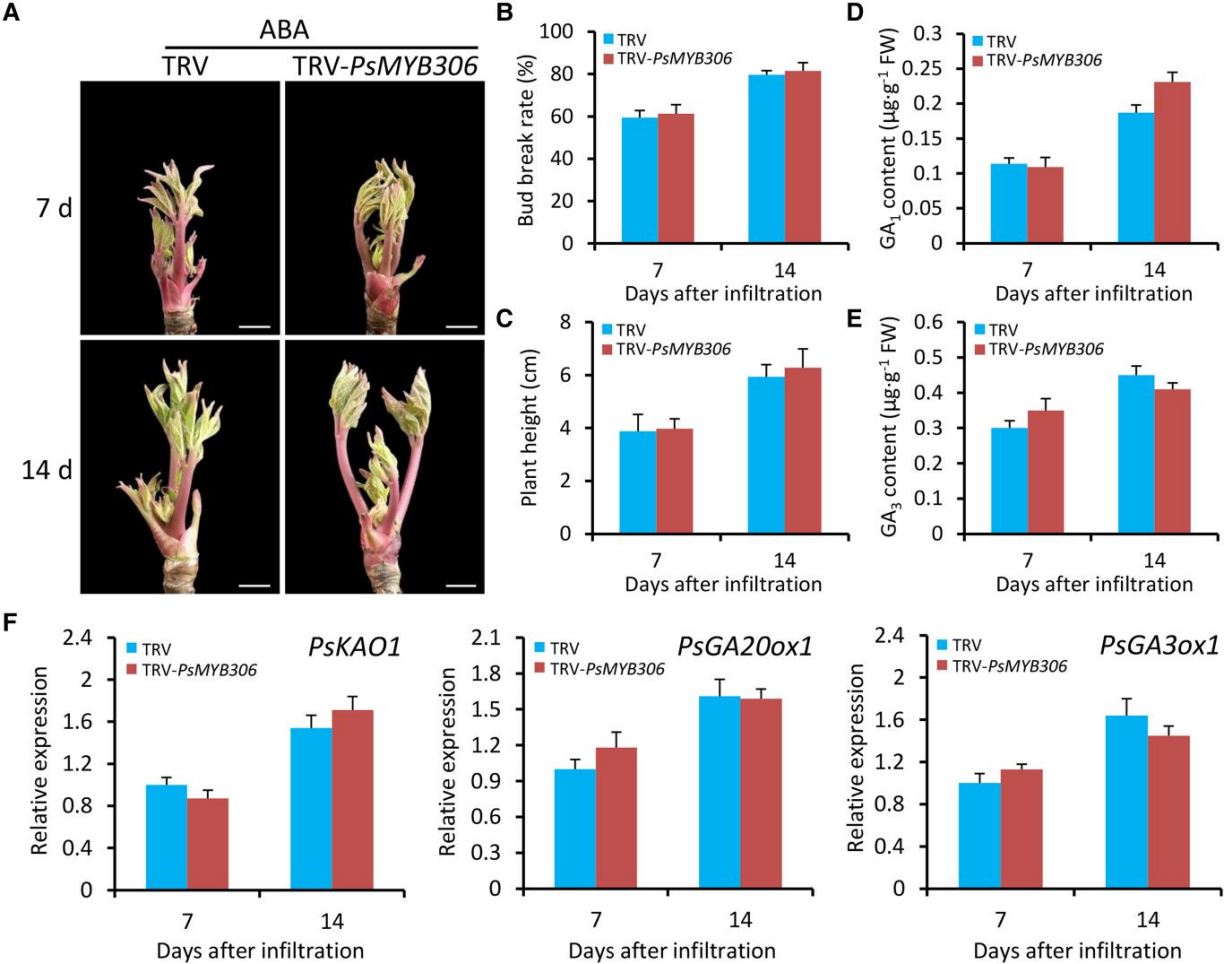
Exogenous application of ABA alleviates the accelerated dormancy release in *PsMYB306*-silenced tree peony buds. **A)** Representative phenotypes of sprouting buds from 1-yr-old grafted tree peony plants infiltrated with TRV empty vector or TRV-*PsMYB306* after treatment with 100 *μ*M ABA. The buds at 3 DAI were used for ABA treatment. Photographs were taken at 7 and 14 DAI. The buds were digitally extracted for comparison. Scale bars = 0.8 cm. Bud break rates **B)** and growth heights **C)** of ABA-treated TRV empty vector- and TRV-*PsMYB306*-infected tree peony plants at 7 and 14 DAI. Contents of bioactive GAs GA_1_ and GA_3_**D, E)** in the ABA-treated buds infected with different TRV constructs at given time points. **F)** RT-qPCR analysis of expression levels of several GA biosynthetic genes, including *PsKAO1*, *PsGA20ox1*, and *PsGA3ox1*, in TRV empty vector- and TRV-*PsMYB306*-infected tree peony plants exposed to ABA treatment at given time points. *PsActin* was used as a reference gene. Error bars represent Se of the mean from 3 biological replicates.

To test the above hypothesis, we investigated the effect of exogenous ABA treatment on dormancy release in empty vector– and TRV-*PsMYB306*-infected tree peony buds under low temperature. At 7 and 14 d after infiltration, it was found that the treatment with ABA reduced the growth of *PsMYB306*-silenced buds, which was similar to that of empty vector–infected buds (Fig. 7A). Similar bud break rates and plant heights were detected in *PsMYB306*-silenced and control buds after ABA treatment at given time points (Fig. 7, B and C). Application of ABA resulted in no significant alteration in the contents of bioactive GAs GA_1_ and GA_3_ in tree peony buds with *PsMYB306* silencing and nonsilencing (Fig. 7, D and E). At the transcript level, correspondingly, the abundances of GA biosynthetic genes *PsKAO1*, *PsGA20ox1*, and *PsGA3ox1* in the buds infected with TRV-*PsMYB306* were almost identical to those in the control buds (Fig. 7F). These data demonstrate that ABA treatment imposes an inhibitory influence on GA production, thereby leading to an alleviation of accelerated cold-induced bud endodormancy release.

### PsMYB306 is involved in ABA biosynthesis by specifically activating the transcription of *PsNCED3*

**Figure 8.**
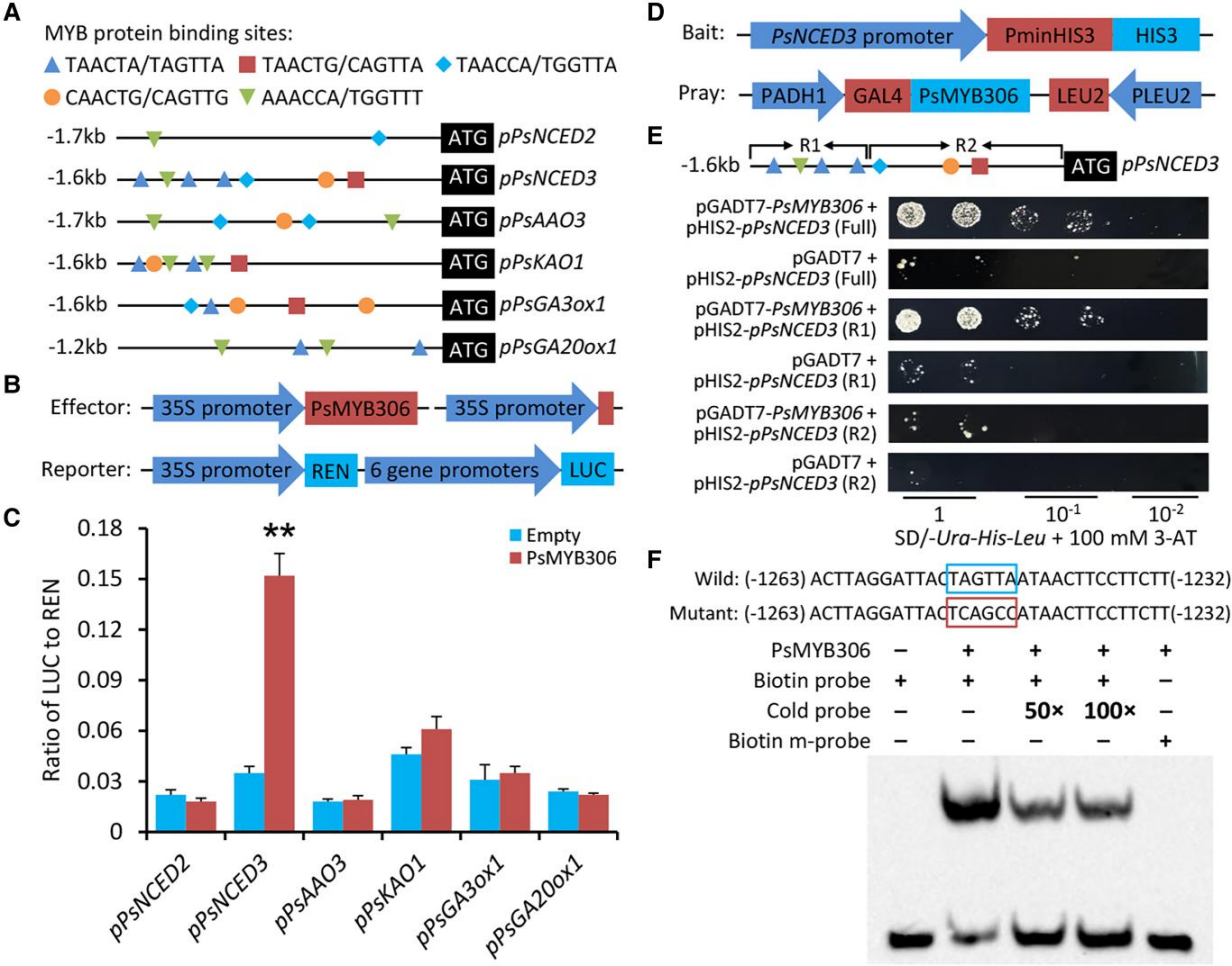
PsMYB306 directly transactivates the promoter of *PsNCED3*. **A)** Graphic representation of the promoters of *PsNCED2*–*3*, *PsAAO3*, *PsKAO1*, *PsGA3ox1*, and *PsGA20ox1* upstream of their coding sequences. Different MYB protein binding sites are marked by regular triangles, squares, diamonds, circles, and inverted triangles. **B)** Schematic diagrams of the effector and reporter constructs for a dual-luciferase reporter system. REN, *Renilla* luciferase; LUC, firefly luciferase. **C)** Dual-luciferase assay of the *PsNCED2*–*3*, *PsAAO3*, *PsKAO1*, *PsGA3ox1*, and *PsGA20ox1* promoters with PsMYB306. The activation activity was evaluated through a LUC/REN ratio. **D)** Schematic diagrams of the bait and pray constructs for yeast 1-hybrid assay. **E)** Growth of yeast cells transformed with the bait and pray constructs on the SD/-*Ura-His-Leu* plates with addition of 100 mM 3-AT. Different dilutions of yeast cells were compared. The R1 and R2 regions are shown in the complete (full) promoter of *PsNCED3*. **F)** EMSA of PsMYB306 binding to the biotin-labeled probe. The predicted WT binding motif (wild) and the mutant one are shown in the box. Nonlabeled probes (cold) at 50- and 100-fold concentrations were referred to as competitors. Error bars represent the Se of the means from 3 biological replicates. Asterisks suggest statistical significance as evaluated by Student's *t* test (**P < 0.01).

To identify the downstream target genes of PsMYB306, the possible MYB binding sites in the promoter regions of genes with variable expression in the VIGS assay were evaluated. A previous publication has reported that Arabidopsis MYB96, a homolog of PsMYB306, could recognize multiple DNA motifs with the consensus sequence HAACYR (or YRGTTD in the opposite strand) for the binding (Seo et al. 2011). Some of these motifs were identified in the promoters of those differentially expressed ABA and GA biosynthetic genes. Of them, the promoter of *PsNCED3* harbors more binding sites than the others (Fig. 8A; Supplementary Fig. S4). A dual-luciferase reporter system was used to examine the potential interaction between PsMYB306 and complete promoters of *PsNCED2–3*, *PsAAO3*, *PsKAO1*, *PsGA20ox1*, and *PsGA3ox1*. PsMYB306 was found to only activate the *PsNCED3* promoter, causing a 4.3-fold rise in the activities of firefly luciferase (LUC; Fig. 8, B and C). To validate this activation, another reporter system involving a yeast 1-hybrid assay was employed. The complete sequence (full) of *PsNCED3* promoter was divided into 2 distinct regions R1 and R2. The interaction of PsMYB306 with *PsNCED3* promoter (full or R1) enhanced the yeast cell growth on the SD/-Ura-His-Leu medium containing 3-aminotriazole (3-AT). However, a disrupted propagation of yeast cells similar to the controls was observed for *PsNCED3* promoter (R2; Fig. 8, D and E). Given the fact that 3 TAACTA (or TAGTTA) elements were present within the R1 region, we performed an electrophoretic mobility shift assay (EMSA) to verify whether PsMYB306 could bind to this core element. A 32-bp fragment bearing the TAGTTA element was used as a probe, and its mutation was created via single-nucleotide substitution. The EMSA data showed a strong binding of PsMYB306 to the biotin-labeled probe, and the binding signals were attenuated with addition of unlabeled probe. No binding signal was detected in the reaction with the mutant probe (Fig. 8F). These observations indicate that PsMYB306 directly binds to the promoter of *PsNCED3*.

### Silencing of *PsNCED3* accelerates bud dormancy release of tree peony

**Figure 9.**
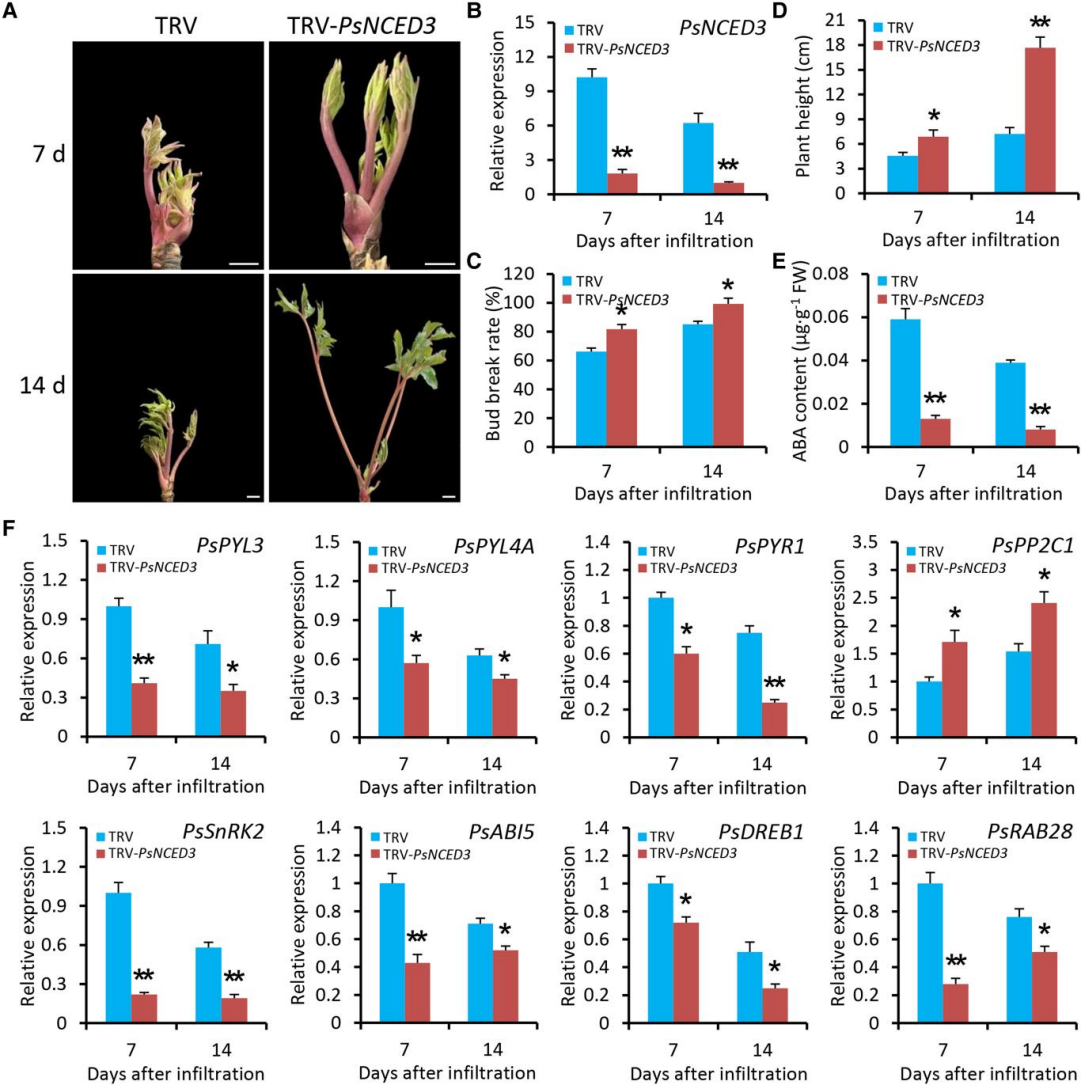
Silencing of *PsNCED3* promotes bud dormancy release in tree peony. **A)** Representative phenotypes of sprouting buds from 1-yr-old grafted tree peony plants infiltrated with TRV empty vector or TRV-*PsNCED3*. The buds at 25 d after chilling were used for infiltration. Photographs were taken at 7 and 14 DAI. The buds were digitally extracted for comparison. Scale bars = 0.8 cm. **B)** RT-qPCR analysis of expression levels of *PsNCED3* in the buds inoculated with various TRV constructs at 7 and 14 DAI. Bud break rates **C)** and growth heights **D)** of TRV empty vector- and TRV-*PsNCED3*-infected tree peony plants at 7 and 14 DAI. **E)** ABA levels in TRV construct-infected buds at given time points. **F)** RT-qPCR analysis of expression levels of some ABA-responsive genes in the buds infected with various TRV constructs at given time points. Expression levels were normalized to *PsActin*. Error bars represent Se of the mean from 3 biological replicates. Statistical significance was verified using Student's *t* test (*P < 0.05, **P < 0.01) and denoted by asterisks.

To further study the function of PsNCED3 in the modulation of bud dormancy, *PsNCED3* was downregulated in tree peony buds under chilling stress through the TRV-VIGS method. RT-qPCR analysis revealed an efficient systemic spread of TRV constructs in the uppermost tissues (Supplementary Fig. S5). The buds infected with TRV-*PsNCED3* exhibited promoted sprouting and growth at various days after infiltration (DAI) compared with empty vector–infected ones (Fig. 9A). The transcription of *PsNCED3* was markedly reduced after the infiltration with TRV-*PsNCED3* (Fig. 9B). Consistent with the phenotypes observed, both bud break rates and plant heights were increased in the buds with *PsNCED3* silencing in comparison with the controls (Fig. 9, C and D). Downregulation of *PsNCED3* led to decreased ABA content in the infected buds (Fig. 9E). Moreover, transcript abundances of a number of ABA responsive genes, derived from RNA-Seq data, were examined. As shown in Fig. 9F, expression levels of *PsPYL3*, *PsPYL4A*, *PsPYR1*, *PsSnRK2*, *PsABI5*, *PsDREB1*, and *RESPONSIVE TO ABA 28* (*PsRAB28*) decreased in *PsNCED3*-silenced buds, whereas *PsPP2C1* was upregulated. These results suggest a negative role of PsNCED3 in regulating bud endodormancy release, which is similar to PsMYB306's role.

## Discussion

**Figure 10.**
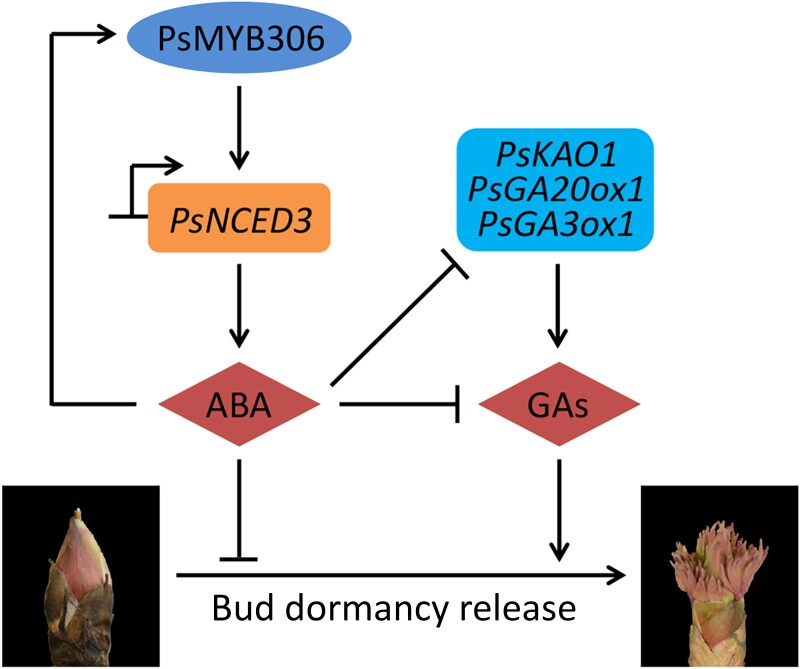
Proposed model for the involvement of PsMYB306 in the regulation of tree peony bud dormancy. PsMYB306 participates in ABA biosynthesis by directly binding to the promoter of *PsNCED3*. ABA is antagonistic to GAs by affecting the transcription of GA pathway–associated *PsKAO1*, *PsGA20ox1*, and *PsGA3ox1*. The treatment with ABA rather than GAs upregulates *PsMYB306* in tree peony buds. PsMYB306 plays a negative role in the regulation of bud dormancy release. Solid lines ending with arrows or short perpendicular lines represent positive or negative regulation, respectively.

Bud dormancy is an efficient strategy for perennial plants to cope with the harsh environment in winter. A full understanding of the regulatory mechanism underlying bud dormancy is crucial to precisely control growth and flowering time in tree peony industry. Although ABA and GAs have been revealed to participate in the regulation of bud endodormancy, how this regulation is fine-tuned by the TFs remains largely unknown. In the present study, we report an important role of a R2R3-MYB TF, PsMYB306, in tree peony bud endodormancy regulation. PsMYB306 was revealed to negatively regulate bud dormancy release by specifically activating the transcription of a key ABA biosynthetic gene *PsNCED3* (Figs. 6 to 8), which was further identified as a repressor of bud dormancy release (Fig. 9). Based on the data presented here, a model was thus proposed for depicting the contribution of ABA-induced PsMYB306-PsNCED3 module to bud endodormancy regulation by integrating the ABA and GA pathways (Fig. 10).

### PsMYB306 acts as a pivotal modulator of bud dormancy in tree peony

Many studies have reported that the members of R2R3-MYB family are implicated in diverse physiological processes, such as plant growth and development (Hu et al. 2020; Du et al. 2022). For instance, PttMYB21a has been found to be involved in secondary vascular tissue development of hybrid aspen (Karpinska et al. 2004). RhMYB108 was shown to function as a positive modulator of flower senescence by perceiving ethylene and jasmonic acid (JA) signals in rose (*Rosa hybrida*; Zhang et al. 2019). The peach MYB10.1 played a role in the vegetative and reproductive development, and its overexpression caused irregular leaf shape and reduced plant height (Rahim et al. 2019). The latest finding showed that OsMYBAS1 regulated rice (*Oryza sativa*) seed germination and seedling establishment under deep-sowing conditions (Wang et al. 2022). Besides, numerous studies have uncovered the close relationship between MYB genes and anthocyanin accumulation in the leaves (Song et al. 2021), petals (Zhang, Xu, et al. 2021), seeds (Gao, Han, et al. 2021), or fruits (Zhang et al. 2023). To date, however, little is known about the transcriptional regulation of bud dormancy by MYB TFs in perennial plants. An in-depth investigation for the bud dormancy–associated TFs has been widely performed via the RNA-Seq method (Gai et al. 2013; Howe et al. 2015; Ionescu et al. 2017; Zhu et al. 2021).

Our RNA-Seq data showed that *PsMYB306* was substantially downregulated in chilling-treated tree peony buds (Fig. 3A), implying its important role in regulating bud endodormancy. This presumption was confirmed by the ectopic transformation and transient VIGS experiments (Figs. 5 and 6). Despite a common understanding that some MADS-box TFs, known as DAMs, emerge as major regulators of the dormancy cycle (Quesada-Traver et al. 2020), our findings provided evidence for the crucial involvement of MYB TFs in controlling bud dormancy. Contrary to the expression of *PsMYB306*, another 2 MYB genes, *PsMYB308* and *PsMYB108*, were found to be upregulated during chilling periods (Fig. 3A). However, their expression was nonsignificantly correlated with the majority of DEGs in the ABA and GA pathways (Fig. 3, B and E). Whether PsMYB308 and PsMYB108 also contribute to the modulation of bud dormancy needs to be investigated in subsequent studies.

Protein modeling analysis reveals that the PsMYB306 harbors 5 key residues in parallel with the Arabidopsis WER structure. Of them, 4 residues seem to be associated with potential DNA binding, while 1 residue, designated L55, was proposed to be responsible for DNA methylation (Fig. 4, B and C). In view of the structural characterization of WER protein (Wang et al. 2020), it is highly likely that this methylation occurs in the core binding element to block the interaction between PsMYB306 and its target DNA. As one of the most abundant epigenetic modifications, DNA methylation has proven to play a pivotal role in the regulation of bud endodormancy (Yang et al. 2021). Studies on sweet chestnut (*Castanea sativa*) showed that the patterns of DNA methylation varied largely during bud set and burst (Santamaría et al. 2009). DNA methylation might participate in the modulation of bud DAM genes under chilling stress (Rothkegel et al. 2017). More recently, long-term chilling was reported to promote endodormancy release and bud break relying on DNA methylation of specific genes in tree peony (Zhang, Xu, et al. 2021). These findings confirm that epigenetic modifications are required for the dormancy control, thereby providing a clue for elucidating the regulatory patterns of PsMYB306 in the bud dormancy.

### PsMYB306 mediates the crosstalk between ABA and GAs during bud dormancy release

ABA and GAs are considered as 2 key phytohormones in regulating the bud dormancy–growth transition. The maintenance of bud endodormancy is dependent on a high level of endogenous ABA, and the overproduction of GAs is required for endodormancy release (Zhuang et al. 2013). The opposite influences of these 2 hormones on bud dormancy have been validated in various plant species, such as poplar (*Populus trichocarpa*; Howe et al. 2015), Japanese apricot (Wen et al. 2016), grapevine (Khalil-Ur-Rehman et al. 2019), pear (Yang et al. 2020), and peach (Hernández et al. 2021). Several studies have demonstrated that application of ABA inhibited while GAs promoted bud burst and shoot growth in tree peony (Zheng et al. 2009; Guan et al. 2019). In support of this notion, the altered production of ABA and bioactive GA_1_ and GA_3_ was found here during chilling-induced bud endodormancy release (Fig. 1, B and C). This finding is in agreement with a previous report, showing a persistent decrease and increase in the levels of ABA and bioactive GA_15_, respectively, in chilling-treated tree peony buds (Zhang et al. 2020). Although a different report showed that ABA content increased initially followed with a drop during bud dormancy transition in herbaceous peony (*Paeonia lactiflora*; Yu et al. 2012) or pear (Li et al. 2018), it is most likely that the low temperature (2 °C) we chose could rapidly initiate ABA degradation to convert maintenance to release of endodormancy. Here, a number of genes in the ABA and GA pathways displayed variable expression through the transcriptome analysis, further confirming the important roles of ABA and GAs in bud dormancy regulation.

As demonstrated by an integrated correlation analysis, *PsMYB306* was found to be positively correlated with *PsNCED3*, a key gene associated with ABA biosynthesis (Fig. 3, B to D). Application of ABA provoked a dramatic increase in *PsMYB306* transcripts (Fig. 4F). This suggests that PsMYB306 probably regulates bud dormancy release by affecting the ABA pathway, which is supported by a direct binding of PsMYB306 to *PsNCED3*'s promoter (Fig. 8). Analogously, PsMYB306's homolog MYB96 was reported to activate ABA biosynthesis by binding to the promoters of *NCED2* and *NCED6*, contributing to the modulation of Arabidopsis seed dormancy (Lee et al. 2015; Lee and Seo 2015). Considering the similarity and difference between ABA-mediated bud and seed dormancy (Wang et al. 2016), a complex regulatory network by PsMYB306 may exist in the dormancy process of various tree peony organs. Apart from ABA, ectopic overexpression and silencing of *PsMYB306* also caused a change in the production of bioactive GAs. However, the evidence showing a direct modulation of GA pathway by PsMYB306 is scarce. We found some correlations of *PsMYB306* with several GA pathway–related genes, but these correlations were not corroborated by the PCA and interaction network analyses (Fig. 3, E to G). Furthermore, the binding tests revealed no interaction between PsMYB306 and the promoters of several GA biosynthetic genes (Fig. 8C). These data suggest that ABA is possibly epistatic to GAs in the process of bud dormancy release, as an alleviation of accelerated dormancy release and increased expression of those GA biosynthetic genes was observed in ABA-treated *PsMYB306*-silenced buds compared with the control ones (Fig. 7). Some similar conclusions have been drawn in previous reports. Yang et al. (2019) found that ABA interfered with the action of PpyGAST1, whose release led to a decrease in *PpyGA20ox2* expression and GA content during bud dormancy transition in pear. ABA-responsive element binding factor ABF3, a member of bZIP family, was characterized as an inhibitor of GA biosynthesis by inducing the transcription of GA-deactivating gene *GA2ox1* (Yang et al. 2021, 2023). It has been recognized that ABA appears to inhibit GA biosynthesis and promote GA catabolism during the dormancy cycle. Thus, we hypothesize that GA may play a dominant role in regulating the bud dormancy transition in comparison with ABA. This may explain an extensive application of bioactive GAs rather than ABA biosynthesis inhibitor, such as fluridone, to break bud dormancy during off-season production of tree peony (Gao, Yuan, et al. 2021; Zhang, Yuan, et al. 2021). In addition, the ABA-dependent plasmodesmata closure is associated with endodormancy establishment by blocking the supply of growth-promoting signals to bud meristems (Tylewicz et al. 2018). Besides the inhibition of GA-induced growth, ABA probably prevents the opening of plasmodesmata required for bud endodormancy release. It has been reported that the elevation of GA content occurred concomitantly with the opening of plasmodesmata during dormancy release (Maurya and Bhalerao 2017). The regulatory role of PsMYB306 in ABA-mediated plasmodesmata closure should be further investigated.

It is noteworthy that PsMYB306 specifically activated the promoter of *PsNCED3* by targeting the TAACTA (or TAGTTA) motif. It has been demonstrated that WER, the protein used for modeling analysis, binds to 2 DNA fragments including aaTgcgGTTgg for GWBSI and aaGTTaGTTga for GWBSII (Song et al. 2011). Both fragments share a core GTT (or AAC) element, which was also found within the PsMYB306-binding motif (Fig. 8F). By comparison, however, some nucleotide discrepancies between WER- and PsMYB306-binding motifs were still observed beyond the core element. Based on phylogenetic analysis, the sequence similarity of PsMYB306 to WER was obviously not the highest (Fig. 4A). Therefore, one of the possible explanations is that the amino acid variations in their R2R3 domains probably result in the differences of DNA binding sites. This is supported by many previous studies in which single or multiple amino acid polymorphisms altered DNA binding specificities of TFs (Bosselut et al. 1993; Ciolkowski et al. 2008; Hichri et al. 2011). Moreover, despite the presence of TAACTA (or TAGTTA) motifs in the promoters of *PsKAO1*, *PsGA3ox1*, and *PsGA20ox1*, we found no direct binding of PsMYB306 to their promoters (Fig. 8C). This indicates that PsMYB306 remains much weaker binding activities to these promoters, probably owing to specific interactions with unknown proteins or other DNA sequences. Additional factors beyond the binding sites are usually required for the protein–DNA binding events in vivo (Yang et al. 2006; Zhou et al. 2015), thereby suggesting that PsMYB306 may have variable preferences for transcriptional activation.

### PsMYB306 may have multiple biological functions by regulating hormonal interplay

The bud dormancy transition is a highly complex process involving various internal and external signals (Liu and Sherif 2019). From the perspective of endogenous hormones, we determined that PsMYB306 affected the ABA/GA balance during bud dormancy release of tree peony in the present study. Notably, it is well recognized that ABA and GAs serve as important signals for influencing plant growth and development. For example, ABA is referred to as a promoter of petal senescence in many ethylene-sensitive plants (Müller et al. 1999; Ji et al. 2022), while GAs can inhibit the senescence progress (Saeed et al. 2014). Similarly, the petal senescence was promoted and suppressed by ABA and GAs, respectively, in ethylene-insensitive gladiolus (Kumar et al. 2014). HOMEOBOX 1 (RhHB1), a HD-ZIP TF, has been reported to mediate the interplay between GAs and ABA during rose petal senescence (Lü et al. 2014). Our latest finding suggests a negative role of OCS ELEMENT BINDING FACTOR 1 (PhOBF1), a member of bZIP family from petunia, in corolla senescence by modulating the GA production (Ji et al. 2023). Our studies here also verified the function of PsMYB306 in the senescence regulation, showing shortened flower longevity in *PsMYB306*-overexpressing transgenic petunia plants (Supplementary Fig. S6). It is speculated that the increased ABA levels by PsMYB306 also take place in the floral tissues. Apart from flower senescence, the antagonistic effect of ABA to GAs has also been revealed during leaf senescence of Chinese flowering cabbage (Fan et al. 2020). Future work will examine whether PsMYB306 is involved in the modulation of leaf senescence.

In addition, the important roles of ABA and GAs in responses to abiotic or biotic stresses should not be ignored. ABA has long been considered as a key hormonal signal induced by several adverse environmental factors, such as cold and drought (Gilmour and Thomashow 1991). Many pieces of evidence have revealed the contribution of ABA to plant tolerance to freezing (Bravo et al. 1998) and drought (Estrada-Melo et al. 2015). Bud dormancy, as manifested by growth cessation, is essential for the acclimation of woody plants to cold and drought in nongrowing seasons. It is an undeniable fact that a sufficient low-temperature accumulation is indispensible to break bud dormancy (Rothkegel et al. 2020). A proper dehydration treatment was reported to accelerate the onset of bud burst (Pellegrino et al. 2020). Furthermore, ABA and GAs have also been demonstrated to be associated with defensive responses to fungal or viral pathogens (Cao et al. 2011; Zhao and Li 2021). How the pathogen attack influences the bud dormancy transition through hormonal signals is still largely unclear. An earlier report showed that the virus-infected buds of *Euphorbia pulcherrima*, an ornamental plant, never became dormant compared with the healthy buds, probably due to virus-induced higher GA levels (Nath and Mandahar 1988). These findings imply the possible involvement of PsMYB306 in host defense against various stressors. Consistent with this hypothesis, we found increased accumulation of TRV RNA1 and RNA2 in *PsMYB306*-silenced buds (Supplementary Fig. S3). Moreover, PsMYB306's homologous protein MYB96 was shown to confer tolerance to drought and osmotic stresses as well as resistance to microbial pathogens (Lee and Seo 2021). The function of PsMYB306 in responses to multiple environmental challenges requires further examination in the future.

It is worth mentioning that the production of several other phytohormones has been altered during chilling-induced bud dormancy transition in tree peony. In detail, JA, salicylic acid (SA), and indole-3-acetic acid (IAA) were reported to accumulate, while a lower concentration of *trans*-zeatin (tZ) was found (Zhang et al. 2020). Our previous studies are partially in accordance with these hormonal variations, showing an increase in JA, IAA, and tZ contents during the initial periods of bud break (Mao et al. 2022). The contradictory observations on tZ levels may suggest the distinct roles of cytokinins during the conversion from dormancy release to bud germination. Particularly, a sharp increase in JA content at the final stage of bud endodormancy is most likely responsible for the anthocyanin accumulation (Zhang et al. 2020), which has been proposed to enhance freezing tolerance in sprouting buds of tree peony (Mao et al. 2022). JA has been demonstrated to control embryonic dormancy in apple by inducing sugar catabolism (Bogatek et al. 2002), suggesting a potential role in the modulation of bud dormancy. The subsequent work should include a study of the responses of PsMYB306 to JA, SA, IAA, and tZ during bud dormancy release. More importantly, whether these hormones are involved in bud dormancy regulation still remains to be investigated.

In conclusion, the data presented here indicate that PsMYB306 functions as a negative regulator of bud endodormancy release by modulating the crosstalk between ABA and GAs. A specific transactivation of *PsNCED3*'s promoter by PsMYB306 was revealed through a set of protein–DNA binding assays. PsNCED3 was further confirmed to participate in bud dormancy regulation. These findings add to the existing theoretical foundation of the regulatory mechanism underlying bud dormancy release. It will be useful to manipulate bud dormancy states through genetic engineering, thereby providing a viable solution to achieve the growth and flowering control in tree peony production. More target genes should be identified using the combined multiomics and VIGS approaches, which will help unravel the whole regulatory network of bud dormancy by integrating different hormonal and environmental signals in tree peony.

## Materials and methods

### Plant materials and growth conditions

Tree peony (P. suffruticosa) cultivar ‘Yulouchun’ was used as the main experimental material in this study. Five- or 1-yr-old grafted tree peony plants were potted with the soil mixture containing peat moss and perlite (2:1, by vol). The plants were grown in the natural environment until the beginning of November when the bud endodormancy was initially established (Huang, Zhu, et al. 2008; Zhang et al. 2015). After removing dry shoots and leaves, they were then transferred to a cold room and treated with chilling stress at 2 °C in the dark. The apical buds at different time points after treatment were harvested for the measurement of hormone content. The chilling-treated buds from 5-yr-old plants at 0, 15, and 30 d were used for the RNA-Seq analysis. The buds from 1-yr-old plants at 25 d after chilling treatment, near the time of a complete endodormancy release, were used for the VIGS experiment. The seeds of petunia (P. hybrida) cultivar ‘Mitchell Diploid’ were purchased from Goldsmith Seeds Inc. (Gilroy, CA, USA). They were sown in the pot containing the same soil mixture. The younger petunia leaves were harvested before flowering period for Agrobacterium-mediated genetic transformation. The tree peony upon VIGS and transgenic petunia plants were maintained in a growth chamber at 20 °C with a 16/8-h light/dark photoperiod. For expression analysis of PsMYB306 in response to exogenous hormones, the buds prior to chilling treatment were sprayed by 100 μM ABA and 100 μM GA_3_. For tissue-specific expression analysis of PsMYB306, the uppermost young leaves and stems, newly grown roots, and floral tissues (sepals and petals) at anthesis in April as well as the mature buds and seeds in October were used.

### Detection of endogenous hormones

Tree peony buds treated with chilling or infected with TRV constructs and the leaves from transgenic petunia plants were collected for the determination of hormone levels as previously described (Ji et al. 2022). Approximately 0.5 g of samples was freeze dried and extracted with 80% (v/v) methanol and 1 mM butyl hydroxytoluene. The extract was transferred into a tube with 20 mg of PVPP and fully mixed. The mixture was centrifuged at a low temperature at 5,000 rpm for 20 min. Under the condition of 40 °C, the extract was almost concentrated into a water phase containing ammonia. After filtration with a 0.45-µm filter, the sample was dried through a vacuum freezing method. The particles were then dissolved in 50% (v/v) methanol and analyzed by HPLC using an Agilent chromatograph (Model 1100, Agilent Technologies, Santa Clara, CA, USA). The detection wavelength used in this experiment is 210 to 280 nm, and the flow rate is 1 mL/min. Three biological replicates were used for this assay. The standards were purchased from Sigma-Aldrich (St Louis, MO, USA). The peak areas of bud sample and standard sample were compared to quantify the levels of ABA and GAs. Three biological replicates were used for each hormone measurement.

### RNA-Seq and data processing

Six tree peony buds randomly harvested from 3 5-yr-old grafted plants were pooled at each of the 3 chilling stages from S1 to S3. Three biological replicates of every pool were used to prepare the RNA samples. RNA extraction was conducted using an RNAprep Pure Plant Plus Kit DP441 (TIANGEN, Beijing, China). The purity and concentration of RNA samples were examined using a NanoDrop ND-2000c Spectrophotometer (NanoDrop Technologies, Wilmington, DE, USA). The library construction and Illumina RNA-Seq analysis were performed using an Illumina HiSeq2000 equipment at Gene Denovo Biotechnology Co., Ltd (Guangzhou, China). The raw data files are brokered to the NCBI's Sequence Read Archive (SRA) database with an accession number PRJNA992915. To ensure the quality of sequencing data, the original reads were filtered to obtain the clean reads using Perl script tool. The clean reads were further assembled to the unigenes (≥200 bp) through Trinity software (version 2.1.1; Grabherr et al. 2011) and TIGR Gene Indices clustering tools (version 2.1; Pertea et al. 2003). The sequences of unigenes were used for gene expression and functional investigation in this study (Supplementary Data Set 1). All the unigenes were annotated against the Nr, KEGG, COG, and Swiss-Prot databases. Transcript levels of unigenes were quantified based on the reads per kilobase of exon model per million mapped reads (RPKM) through Cufflinks program (version 2.1.1; Trapnell et al. 2012). DESeq2 analysis was performed to identify the DEGs between samples. The significant DEGs were identified using the following criteria: fold change ≥2.0 and false discovery rate (FDR) ≤0.05 through edgeR package (version 3.12; Chen et al. 2014). Violin plots were constructed using R scripts. For the pathway annotation, the DEGs were searched against the KEGG database (http://www.genome.jp/kegg/; Wixon and Kell 2000) using FDR ≤0.05 as a cutoff.

### Subcellular localization and transactivation assay

The coding sequence of PsMYB306 without the termination codon was constructed into the pCAMBIA2300-GFP vector between KpnI and BamHI sites to generate the recombinant construct. The recombinant construct and empty vector pCAMBIA2300-GFP were transformed into Agrobacterium tumefaciens GV3101 cells by electroporation, which were then infiltrated into the young leaves of 4-wk-old N. benthamiana plants. The N. benthamiana plants were incubated at 25 °C in the dark for 48 h, and the nuclei were stained with DAPI. H2B-mCherry was used as a reference for nuclear localization. The fluorescent signals were observed under a confocal laser scanning microscope (Leica SP8, Solms, Germany). GFP and mCherry were excited using 488 and 561 nm lasers and detected after passing through 500 to 560 nm and 590 to 620 nm band-pass filters, respectively. At least 5 leaves were observed for each infiltration. For the determination of transcriptional activity, the coding region of PsMYB306 without the termination codon was cloned into the NdeI–BamHI sites of pGBKT7 vector (Clontech, Palo Alto, CA, USA) to generate the PsMYB306-BD construct. The pCL-1 construct and empty vector were used as the positive and negative controls, respectively. They were transformed into the Saccharomyces cerevisiae strain AH109 cells, which were grown on the defective media SD/-Trp and SD/-His-Trp. The yeast growth was analyzed as previously described (Gan et al. 2021).

### RT-qPCR assay

Total RNA was extracted using the method as mentioned above. After checking RNA purity and yield, first-strand cDNA was synthesized with premix reagent (TIANGEN, Beijing, China) for reverse transcription. qPCR was performed using SYBR Premix Ex Taq II Kit (TaKaRa, Dalian, China). This reaction was performed in a LightCycler instrument (Roche Diagnostic, Basel, Switzerland). Expression levels were normalized to PsActin (Gao et al. 2023) and ELONGATION FACTOR 1-ALPHA (PhEF1α; Mallona et al. 2010) for tree peony and petunia, respectively. Relative expression data were analyzed using the 2^−ΔΔCT^ calculation method. The primers used for qPCR analysis were designed by Primer Premier 5.0 software, and they are listed in Supplementary Table S6. Three biological replicates were used for each expression assessment.

### Generation of transgenic petunia plants

To generate a construct for stable transformation in petunia, the coding region of PsMYB306 was introduced into the pGSA1403 vector between the sites of XhoI and SacI. The recombinant plasmid was transformed into A. tumefaciens strain LBA4404 cells. The stable transformation in petunia was conducted using Agrobacterium-mediated leaf disk method as previously described (Ji et al. 2023). The young leaves of ‘Mitchell Diploid’ petunias were cut into squares (1 cm × 1 cm) and inoculated with Agrobacterium harboring the recombinant plasmid. The positive transgenic plants were selected on the MS medium supplemented with 100 mg/L kanamycin. At the 4- to 6-leaf stage, a regular PCR amplification was performed to confirm the integration of PsMYB306 into the petunia genome. At least 10 positive transformants were obtained through the PCR method. Simultaneously, RT-qPCR was used to examine transcript levels of PsMYB306 in the leaves from WT and transgenic petunia lines. Three lines with relatively higher transcription of PsMYB306 were selected for a continuous cultivation to obtain homozygous seeds, which were used for subsequent germination and growth analysis.

### VIGS assay

This assay was performed as previously described (Mao et al. 2022). A 264-bp fragment of PsMYB306 and a 348-bp fragment of PsNCED3 were inserted into the SacI–XhoI sites of TRV2 vector. The resulting plasmids were transformed into A. tumefaciens strain GV3101 cells, which were cultured in LB medium containing 40 mg/L kanamycin at 28 °C for 48 h. The culture was then centrifuged at 4,000 rpm for 15 min at room temperature. The harvested cells were resuspended with the infiltration buffer containing 200 μM acetosyringone, 10 mM MES, and 10 mM MgCl_2_, with the concentration adjusted to OD_600_ of 0.6. After a gentle shaking for 3 h, the Agrobacteria transformed with TRV1 and TRV2 constructs were mixed at 1:1. At 25 d after chilling treatment, the bud scales from 1-yr-old grafted tree peony plants were partially removed to facilitate the entry of inoculum. The plants were placed upside down into the Agrobacterium mixture to make the buds fully submerged. The infiltration was carried out under a vacuum at 0.7 MPa for 20 min. Next, the infiltrated plants were transplanted into the pots filled with soil mixture. To promote the viral replication, the plants were exposed to low temperature at 10 °C for 2 d in the dark before the dormancy release test. Three plants were used for each infiltration.

### Isolation and identification of *PsMYB306*

The PsMYB306 cDNA sequence harboring a complete 981-bp coding region was isolated from the RNA-Seq data in chilling-treated tree peony buds. Its nucleotides were translated into corresponding amino acids using ExPASy tool (http://web.expasy.org/translate/). A phylogenetic tree was constructed using MEGA software (version 4.0.2). The conserved R2 and R3 repeats were determined based on a previous report (Bedon et al. 2007). For protein modeling analysis, the homologous structures (Protein Data Bank: 6KKS, 1H89, 1GV2, and 1MSE) of PsMYB306 were identified from the RCSB Protein Data Bank (https://www.rcsb.org/). According to the sequence alignment by Chimera software (version 1.12; Pettersen et al. 2004), the modeling process based on R2 and R3 domains was performed using the Modeling server (version 9.20). Six helixes were included in the R2R3 domains. The 6KKS structure of Arabidopsis WER was used as the template. The model was evaluated through Discrete Optimized Protein Energy (DOPE) values and GA 341 scores and finally visualized using PyMOL tool (version 2.5.4). Five putative conserved residues associated with DNA binding or methylation within the PsMYB306 structure are shown as sticks.

### Measurement of chlorophyll content

Total chlorophyll content was measured according to a previously described method (Yin et al. 2010). About 0.5 g of fresh petunia leaves, collected from the seedlings germinated on MS plates, was ground to fine powder using a mortar and pestle with liquid nitrogen. The powder was placed into a 15-mL centrifuge tube. The extraction of chlorophyll was conducted with 10 mL of acetone:anhydrous ethanol (1:1, by vol) mixture at room temperature in the dark for 24 h. The contents of chlorophyll a and b were analyzed using a Beckman DU-730 UV-visible spectrophotometer (Beckman Instruments, Palo Alto, CA, USA). The spectrophotometric absorbance was determined at wavelengths of 663 and 645 nm for chlorophyll a and b, respectively. Three biological replicates were used for the measurement.

### Dual-luciferase assay

This assay was conducted using a previously described protocol (Sun et al. 2019). To generate the effector construct, the ORF of PsMYB306 was cloned into the pGreenII62-SK vector between XhoI and KpnI sites. To generate the reporter constructs, the promoter regions of PsNCED2, PsNCED3, PsKAO1, and PsGA3ox1 were ligated into the pGreenII0800-LUC vector between the SalI and BamHI sites, while the promoters of PsAAO3 and PsGA20ox1 were inserted into the SalI–SmaI and PstI–BamHI sites, respectively. The transcription of PsMYB306 was driven by CaMV 35S promoter in the effector. The expression of LUC and Renilla luciferase (REN) was activated by 6 gene promoters and CaMV 35S promoter, respectively, in the reporters. The specific primers used are shown in Supplementary Table S6. The recombinant plasmids were transformed into A. tumefaciens strain GV3101 cells. The transformed Agrobacteria were used to coinoculate the young leaves of 4-wk-old N. benthamiana plants. At least 5 leaves were used for each coinoculation. The LUC and REN activities were measured using a luminometer (Männedorf, Switzerland) and shown as the LUC/REN ratio.

### Yeast 1-hybrid assay

This assay was performed according to a previously described method (Ji et al. 2023). The whole promoter sequence (full) of PsNCED3 was divided into 2 regions (R1 and R2). A 1,600-bp DNA fragment for full, 392-bp for R1, and 1,203-bp for R2 were introduced into the NdeI–XhoI sites of the pHIS2 vector, which were referred to as the bait constructs. The complete coding sequence of PsMYB306 was introduced into the EcoRI–SacI sites of the pGADT7-Rec vector harboring the GAL4 activation domain to generate the pray construct. These plasmids were cotransformed into S. cerevisiae strain Y187, whose positive colonies were picked and transferred into liquid medium. LB medium was used to dilute liquid culture to 10 and 100 times. For each dilution, the yeast cells were spotted on the SD/-Ura-His-Leu plates supplemented with 100 mM 3-AT. The cell growth rates were analyzed to assess the binding relationship between PsMYB306 and 2 regions of PsNCED3 promoter.

### EMSA

The EMSA experiment was carried out as previously described (Ji et al. 2023). The complete coding sequence of PsMYB306 was jointed with the pET28a vector between the BamHI and HindIII sites, and the resulting plasmids were transformed into Escherichia coli Rosetta (DE3) cells. Expression of His-tagged PsMYB306 protein was induced by 0.1 mM isopropylthio-β-galactoside. The induced protein was released from the bacterial cells by an ultrasonic treatment and further extracted and purified using the HisTrap HP column. The WT and mutant probes labeled with biotins were synthesized and annealed based on a 32-bp DNA fragment in the promoter of PsNCED3. The WT probe with no labeling was used as the competitor (Supplementary Table S6). The protein–DNA interaction was performed using a LightShift EMSA Optimization and Control Kit (Pierce, Thermo Fisher Scientific, MA, USA). The interaction products were separated by SDS-PAGE electrophoresis and transferred to a nylon membrane through a transfer apparatus (Bio-Rad, Hercules, CA, USA). The binding signals were examined using the Chemiluminescent Nucleic Acid Detection Module Kit (Pierce, Thermo Fisher Scientific, MA, USA). Photographs were taken in the Gel Doc XR+ imaging system (Bio-Rad, Hercules, CA, USA).

### Statistical analysis

The significance of difference was examined using Student's t test at P < 0.05 or P < 0.01 through JMP software (version 11.0). Three biological replicates were used for each assay. The correlations between genes or samples were determined based on Pearson's correlation coefficient (r) with SPSS system (version 19.0). The TBtools software (version 1.087; Chen et al. 2020) was used to create the heatmaps. The PCA was carried out using the gmodels package of statistical program R (http://www.r-project.org/) or SIMCA-P software (version 14.1, Umetrics, Umea, Sweden). The interaction network was generated in the R environment (https://www.r-project.org/) with a coefficient of R ≥ 0.5 or R ≤ −0.5 and visualized through Cytoscape tool (version 3.7.0; Luo et al. 2021). The interacting genes with close relationships were regarded as the hub genes.

### Accession numbers

Sequence data from this article can be found in the GenBank/EMBL data libraries under the following accession numbers: *PsMYB306* (OR992014), *PsNCED2* (OR992008), *PsNCED3* (OR992009), *PsAAO3* (OR992010), *PsGA20ox1* (OR992011), *PsGA3ox1* (OR992012), and *PsKAO1* (OR992013).

## Supplementary Material

kiae014_Supplementary_Data

## Supplementary data

The following materials are available in the online version of this article.

**Supplementary Figure S1.** Annotation and evaluation of RNA-Seq data in chilling-treated tree peony buds.

**Supplementary Figure S2.** The nucleotide and deduced amino acid sequences of *PsMYB306*'s coding region.

**Supplementary Figure S3.** Virus accumulation levels in systemically infected leaves with TRV empty vector and TRV-*PsMYB306*.

**Supplementary Figure S4.** The promoter sequence upstream of *PsNCED3*'s coding region.

**Supplementary Figure S5.** Virus accumulation levels in systemically infected leaves with TRV empty vector and TRV-*PsNCED3*.

**Supplementary Figure S6.** Overexpression of *PsMYB306* accelerates petal senescence in petunia.

**Supplementary Data Set 1.** Sequences of all unigenes assembled from RNA-Seq data in tree peony buds.

**Supplementary Table S1.** Statistics of RNA-Seq data set during bud dormancy release of tree peony.

**Supplementary Table S2.** All the unigenes from RNA-Seq data in tree peony buds.

**Supplementary Table S3.** DEGs during bud dormancy release of tree peony.

**Supplementary Table S4.** KEGG pathway annotation of DEGs in tree peony buds.

**Supplementary Table S5.** Differentially expressed TFs during bud dormancy release of tree peony.

**Supplementary Table S6.** Primers used for gene expression analysis and fragment amplification.

## Dive Curated Terms

The following phenotypic, genotypic, and functional terms are of significance to the work described in this paper:

MYB96 Gramene: AT5G62470
MYB96 Araport: AT5G62470

ABF3 Gramene: AT4G34000
ABF3 Araport: AT4G34000

MYB101 Gramene: AT2G32460
MYB101 Araport: AT2G32460

NCED2 Gramene: At4g18350
NCED2 Araport: At4g18350

NCED6 Gramene: AT3G24220
NCED6 Araport: AT3G24220

bud AmiGo: PO:0000055

trans-zeatin CHEBI: CHEBI:16522
