# Supplementary material for: Abscisic acid–induced transcription factor PsMYB306 negatively regulates tree peony bud dormancy release
Source: Plant Physiol. 2024 Jan 11;194(4):2449–71. doi: 10.1093/plphys/kiae014 (PMC10980420; doi:10.1093/plphys/kiae014)
Supplement: kiae014_Supplementary_Data [file kiae014_supplementary_data.zip › Supplemental Data.pdf]

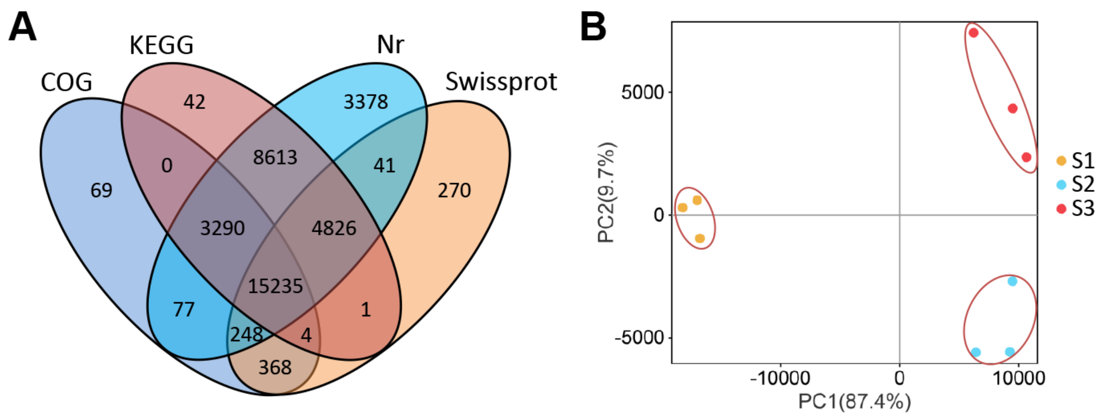

Supplemental Figure S1 Annotation and evaluation of RNA-Seq data in chilling-treated tree peony buds. (A) Venn diagram of unigene numbers annotated by BLASTx with an E-value threshold of 1×10^{-5} against Nr, KEGG, COG, and Swissprot databases. (B) Principal component analysis of RNA-Seq data for each sample of tree peony.

agtgaggaagaagaagacctgtgttgaaaaccatacttatctagggatcaacgttaagaa

atgggtagaccaccttgctgtgataaaatcggagtgaagaaagggccatggactcccgaa

M G R P P C C D K I G V K K G P W T P E

gaagacatcattctggtgtcttacattcaagaacatggaccagggaattggagagccgtt

E D I I L V S Y I Q E H G P G N W R A V

ccaactaatacaggattacttagatgcagcaagagctgcagacttaggtggactaactat

P T N T G L L R C S K S C R L R W T N Y

ctccggcctggtatccgtagaggtaacttcactgatcaagaggagaagatgattatccac

L R P G I R R G N F T D Q E E K M I I H

ctccaagctctttgggaaatagatgggctgccatagcttcgtaccttccccaaagaaca

L Q A L L G N R W A A I A S Y L P Q R T

gataatgatataaagaattactggaatacccatctaaaaaagaagatcaaaaagcttcaa

D N D I K N Y W N T H L K K K I K K L Q

gcaggtgttgatgatgggcacaaccaagatggcctagtttcacaagcaccaatctcaaag

A G V D D G H N Q D G L V S Q A P I S K

ggacagtgggagagaaggcttcaaacagatatccacatggccaaacaagccctttgcgag

G Q W E R R L Q T D I H M A K Q A L C E

gctttgtccttggacaaacctagcaataccatttcacccgagtcgaaaatttgccctaat

A L S L D K P S N T I S P E S K I C P N

tacatcggaccagctctccaatcatctacctatgcctccagtactgaaaacatagcaagg

Y I G P A L Q S S T Y A S S T E N I A R

ttgcttgaaggttggatgagaaattcacccaaatcagcccaaaccaactcagatcagaat

L L E G W M R N S P K S A Q T N S D Q N

tccttcattcacaatccggttccgaccggttccagttccagtgaaggggcactgagtgca

S F I H N P V P T G S S S S E G A L S A

acaactcctgatgctttcgactcgctttttggcttcaattcttccaccaattcggaagcc

T T P D A F D S L F G F N S S T N S E A

tcgcaggccgtatccgctgaggaaactgctaacttcagtactcctgaaactagcctttc

S Q A V S A E E T A N F S T P E T S L F

caagatgaaagcaaaccaaatatggagaaaagtcgtgtcccgctcacattgttagagaaa

Q D E S K P N M E K S R V P L T L L E K

tggctcttcgatgatggtgctgctgctcaagggcaggatgatctaattggtatgccgcta

W L F D D G A A A Q G Q D D L I G M P L

gatggaactgctggtttgttc**tag**aaggagttcaagtttcaacctttgtgtttttcttat

D G T A G L F

Supplemental Figure S2 The nucleotide and deduced amino acid sequences of *PsMYB306*'s coding region. The cDNA sequence of *PsMYB306* harbors a 981-bp open reading frame region encoding a polypeptide of 327 amino acids. The italic bold font and bold font in square denote the start and stop codons, respectively. The font shaded in grey indicates the conserved R2 and R3 domains.

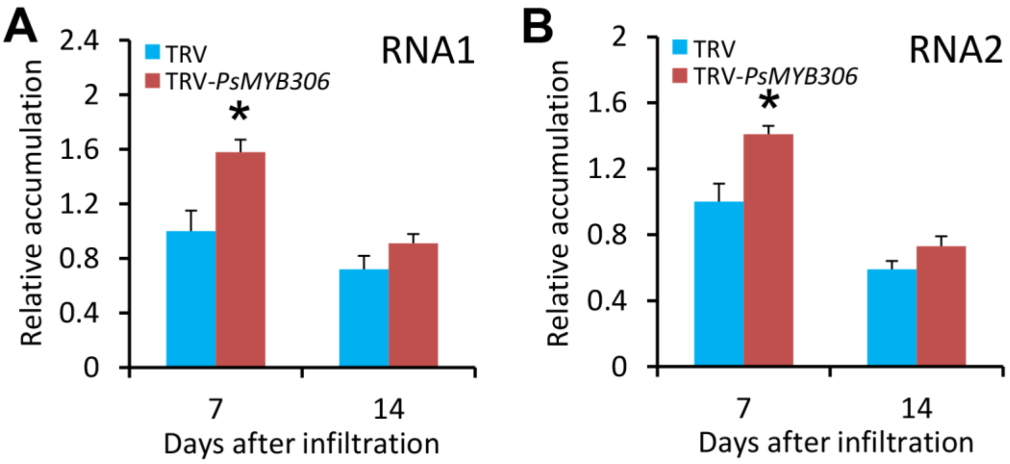

Supplemental Figure S3 Virus accumulation levels in systemically-infected leaves with tobacco rattle virus (TRV) empty vector and TRV-*PsMYB306*. Reverse transcription quantitative PCR analysis of accumulation levels of TRV RNA1 (A) and RNA2 (B) in upper leaves from tree peony plants infiltrated with empty vector or TRV-*PsMYB306*. The leaves at 7 and 14 days after infiltration were harvested for TRV accumulation analysis. Accumulation levels were normalized to *PsActin*. Error bars represent standard error of the mean from three biological replicates. Statistical significance was determined using Student's t test ($^{*}P < 0.05$, $^{**}P < 0.01$) and shown as asterisks.

GCTTCTGAAAGCTATGTCCAGCTAATCTAAATATCGGGGTGCTAAGATAAGCCCATTTCTAG
GTATGTGATTCTTTTATAATTTTTCTTTAGTCATATATCAATGTTTCGATTTAAAAATATTC
TTCTACATGTGTTGATTCTAATACCTAGATTGAGCATGATTTTAGGGTTTATTCAAATTCAA
TCTTTATGTTTCTTGTGTATGAGAGCATGCTAGATTTTATACGATAATTTTCTTGGATACTT
ATTTTGCCAATTGGATTTAAGCATTGCGGTTTGGAACGATGCTTAACTATTCATTTATTCTT
GGGAATACACATTAATTATATTGCATATATTTGAAGATATTTTATGTTGTTGTGTAATAAAA
TGAATGGTTTATATTTTACAACTTAATTGTGCAATTTCTTTTGTTGTTGGTAACATATGTCA
TTGATGACTAAATACAGTCAGATAGAATTGCATATCTAGGTAGGCGGAGGACTTAGTTACCC
TAGTCACTATCTTTATTTGATTAAAACTTTATCACTTTAATTTTATGCATCCATATTATTGA
AAATCATAAAAATATCTACCTTATCTTTATATTACTTATTCTATTACTTAGGATTACTAGTT
AATAACTTCCTTCTTCTTCTTCCTTGTTGATTTGATACTTTTTCCATGTACATATGCTACGA
TTGTCTTATTTGGTTACTTTAGACAAGTAAAACTCCAAGTCTCTCTATTATAATTCACACCA
TCATACACACCACCCAATACACCCTGAATCCCCCATATTTACAAAACCTTTTATAGCCAATA
CTGATTCTCACACCGAAGTTGAATTGGACGAGTTCACGTCGTTCACCTCTGAAGTGATTGTT
TACTGAATTTGCAGGAAGCGGATTTCATCAAGCGCGTTGTCAGATTGTTTGGTTCCATCATC
TCATTGTACAAATTTTCCCTCGTACATCCAATAGGTTGAAGTATCATTCAGGGAGGATCATC
CCTCGAACACAGAATGCCTCAAACCTAATGTCGTTTATCTATTAAGTGATTTTTTGAAGCTT
TTTCAAGGAACTCTAATTACCAATATTGCTCTGACATGAACAGATCACTCCCCACTATCCTC
ATTTATTTCCACTGTCAGACCCCCATAGGCCTACACACGTAATATTTTGGAAGTAAAATTTT
CTCTCCCTCACCCTTTGTTTTTAAGATTCCCAGAAGCTTTATGCAGGCCTCATGATCTTTTT
TCCCTCACCCAGTTGATAAGCCCTCTCAACACTAAAATACACAACCCAAATTTGTTATCCCC
TTATGGTGATTTTCTAAATTATGAATCTAGTCTGCTTTTATGTTTTCCCAGCTGTAGGCGTA
CAACTATCAGTGCGCAAGTGACCAAACAGTCACATTAGAGTACCACCTGCAATACAATACAG
TTAATTAAGGCCGACGCTATTGATCACCAGGAGAAACTCCATGTTTCACCAAAATTTTAATA
TTTAAAACAACATATTTCTTGTGTTATATTTCACCAAATTAATTATAATTTTAAAATGTGTT
GGGATTAAAGTATAATTAAGAAAAATATTAATATAACATATTATCTTTTTTTACTTACATAT
CGTGTCGTCCACCACTCCACCCTTTTTCTATGCTCTGGCGATATCACCGAAGAAAAAAAGCA
AACATATTCTTGTTTTCTCTCCCAATATCACTGAATGTGTTGAATCTTGCACAGGTACCAAT
TTCTTTCATTGCACTTCGGTCAAACTCGCTTTTCTTCTCTCATTCTCACTTACTTTTTTCTC
TGAAAGGAACCAAAATCATCATTTTCCCCCTAGACTCTCACTGGTGGATTCGCTTTGGAAAC
AGAGCA**ATG**GCTTCGTTGCTA

Supplemental Figure S4 The promoter sequence upstream of *PsNCED3*'s coding region. The putative MYB protein binding motifs are marked in different colors. Of them, orange colors indicate the predicted binding motifs of PsMYB306. The bold font denotes the start codon of translation. The probe used for electrophoretic mobility shift assay is marked in square.

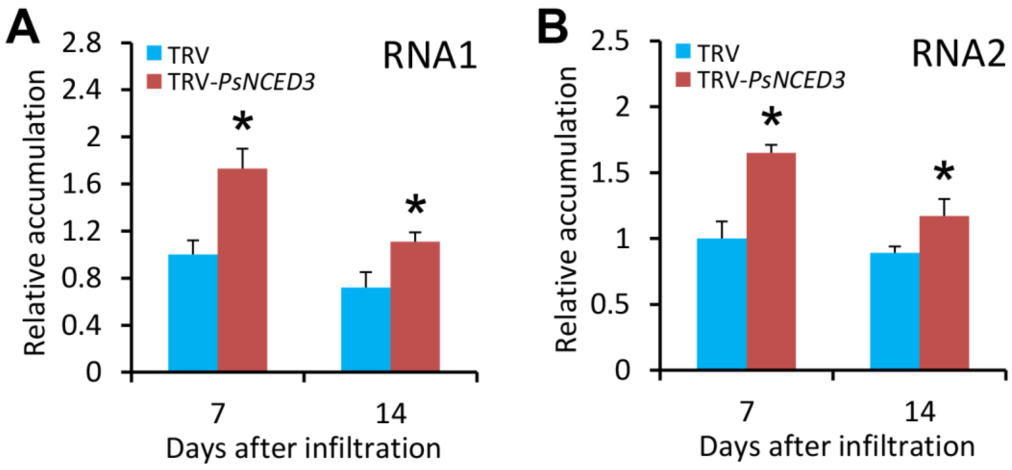

Supplemental Figure S5 Virus accumulation levels in systemically-infected leaves with tobacco rattle virus (TRV) empty vector and TRV-*PsNCED3*. Reverse transcription quantitative PCR analysis of accumulation levels of TRV RNA1 (A) and RNA2 (B) in upper leaves from tree peony plants infiltrated with empty vector or TRV-*PsNCED3*. The leaves at 7 and 14 days after infiltration were collected for TRV accumulation analysis. *PsActin* was used as an internal control. Error bars represent standard error of the mean from three biological replicates. Significance of difference was verified using Student's *t* test (*$P < 0.05$, **$P < 0.01$) and shown as asterisks.

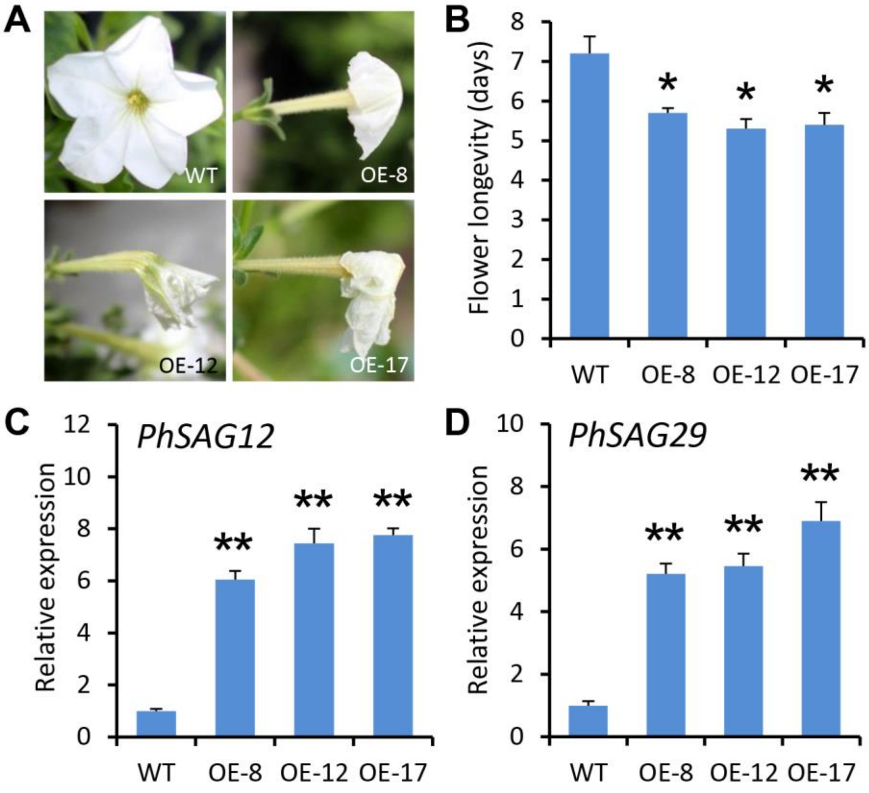

Supplemental Figure S6 Overexpression of *PsMYB306* accelerates petal senescence in petunia. (A) Representative phenotypes of attached flowers from wild-type (WT) and *PsMYB306*-overexpressing (OE) transgenic petunia lines at 6 days (D6) after anthesis. (B) The longevity of attached flowers from WT and transgenic petunia plants. Ten flowers from each of three different plants for each line were counted for longevity evaluation. Flower longevity was determined as the duration from full petal opening to its complete wilting. Relative expression levels of two senescence marker genes *PhSAG12* (C) and *PhSAG29* (D) in the attached flowers from WT and transgenic petunia plants. The flowers at D6 after anthesis were harvested for reverse transcription quantitative PCR analysis. *PhEF1α* was used as an internal control. Error bars represent standard error of the mean from three biological replicates. Asterisks indicate statistical significance as evaluated by Student's *t* test (*$P < 0.05$, **$P < 0.01$).
