# Supplementary material for: Abscisic acid–induced transcription factor PsMYB306 negatively regulates tree peony bud dormancy release
Source: Plant Physiol. 2024 Jan 11;194(4):2449–71. doi: 10.1093/plphys/kiae014 (PMC10980420; doi:10.1093/plphys/kiae014)
Supplement: kiae014_Supplementary_Data [file pp.01764.2023-s05.pdf › pp.01764.2023-s05.pdf]

Plant Physiology®

# An abscisic acid-induced transcription factor, PsMYB306, negatively regulates tree peony bud dormancy release.

Yanping Yuan, Lingling Zeng, Derong Kong, Yanxiang Mao, Yingru Xu, Meiling Wang, Yike Zhao, Cai-Zhong Jiang, Yanlong Zhang, and Daoyang Sun

**Review Timeline:**

| | |
|---|---|
| Submission Date: | 11-Aug-2023 |
| Editorial Decision: | 01-Oct-2023 |
| Resubmission Received: | 08-Nov-2023 |
| Accepted: | 02-Dec-2023 |

01-Oct-2023

Prof. Daoyang Sun
Northwest A&F University
College of Landscape Architecture and Arts
Yangling, Shaanxi Province 712100
China

RE: An abscisic acid-induced transcription factor, PsMYB306, negatively regulates tree peony bud dormancy release.

Dear Dr. Daoyang Sun:

Thank you for giving us the opportunity to review your manuscript for publication in the Plant Physiology. The reviewers have found the manuscript of interest, but have raised a number of concerns that will require your attention. As these concerns are of some gravity and will entail substantial changes to the manuscript, you are likely to need additional time to address them. I must therefore reject the manuscript in its present form. Nonetheless, if you are able to address the points raised in review, I would welcome your resubmission of a manuscript that incorporates your responses.

In particular, I direct your attention to the following comments for consideration in a resubmitted manuscript.

Please, consider describing in detail the plant samples used in all experiments, including the samples collected for RNASeq. Additionally, it is essential to clarify the experiments carried out by semi-quantitative RT-PCR and RT-qPCR, as well as normalizing expression using appropriate reference genes, instead of rRNA.

Please note that any resubmission will be treated as a new entry to the system and is likely to go out for review.

If you do have any queries or concerns, you are welcome to contact me in the first instance.

Thank you again for allowing Plant Physiology to review your work. We look forward to hearing from you soon.

Sincerely,

Board of Editors, Plant Physiology

------------------------------------------------------------------------------ Reviewer comments:
Reviewer #1 (Comments for the Author):

I have many comments and suggestions in the marked up manuscript. Overall, the concept was interesting and based on reasonable data. I understand that the authors want to hold on to the idea of an advancement in the field, but really, we have known for a long time that manipulating ABA and GA levels will effect bud dormancy. We have known also for quite some time that related members of the MYB family alter ABA levels by binding to the promoters of and enhancing expression of NCEDs and thus increasing ABA production. Thus, the results, although novel for tree peony, are not particularly surprising. That said, this is a solid bit of work. It does add to the base of knowledge on dormancy by confirming the solid hypothesis that MYB306 is differentially expressed (down-regulated) during endodormancy breaking, and plays a direct role in reducing the expression of an NCED known to be involved in ABA level maintenance.

There is one significant criticism though, and that was the use of semi-quantitative PCR to examine the expression of multiple genes. This is unacceptable in the current day in age where qRT-PCR is available. Unless data is provided that shows that the PCR reactions were stopped for analysis during the logarithmic stage, the data is completely unreliable. Thus any data that used this technique needs to be reassessed using appropriate methods. As some of this data (as best I can tell from the methods section and the figure legends) are key to the results and discussion, several of the key "findings" are not publishable. I understand the expense of doing so many qRT-PCR runs, but it is unavoidable. One option would be to collect samples for analysis at various cycles and demonstrate that the results are in the logarithmic phase for both the test gene and the internal control.

Reviewer #2 (Comments for the Author):

The manuscript contributes to elucidate bud dormancy regulation in tree peony associated with ABA and GAs. For this purpose, a complete set of techniques (Illumina RNA sequencing approach using buds at different times of a chilling treatment; protein

modelling, overexpression study; gene silencing; in vivo and in vitro binding assays) is conducted.
The manuscript is a nice piece of work, well designed and well written. The methods used are adequate, and results and discussion are clearly exposed. The conclusions are justified based on the results obtained. Overall, the data presented highlights PsMYB306 as a negative regulator of bud dormancy release by modulating the crosstalk between ABA and GAs. Moreover, a specific transactivation of PsNCED3's promoter by PsMYB306 was observed. In my opinion, this type of work brings novelty to the understanding of the regulatory mechanism underlying bud dormancy release, and this is reflected adequately in the discussion's section. The genes identified by this type of approach should have a broader significance by performing multi-omics studies.
Nevertheless, I have some questions at different sections of the manuscript.
In the Introduction and along the entire work, genes should be spell out to describe to the reader what pathways are described.
In the Methodology and Results described:
-The RNAseq samples used where from the buds at 0(S1), 15 (S2) and 30 (S3) days after chilling treatment. Could you explain why? How are the morphology of these samples? Is there any picture about it? How much time does it take for a bud to release dormancy in natural conditions? Without the artificial chilling treatment? Is there a control where no chilling treatment was used? In my opinion, this should be the control of the whole experiment, not only the 0 chilling treatment, but the one in normal conditions to compare with the S2 and S3, for example.
Figure 1: In A, the image is the pot of a petunia where you can observe the buds. However, in Figure 5, the OE of PsMU306 is shown at 10 days after sowing (DAS). The relative expression levels (B) is indicated in young leaves at 10DAS, however the whole story goes about the bud dormancy. Why these experiments shown are not done in buds but in young leaves?? How are the buds after 10DAS? Could we see a picture of that? The G pictures, are done at 50DAS?, where and how are the buds over there? If the leaves are already formed, there is no dormancy over there. So, the whole point to show the role of MYB306 and the subsequent genes which it interacts in dormancy is not really provided, or am I wrong?

Figure 2: should be explain the genes described, especially when along the text where not explained, like e.g. PYL/PYR/RCAR. The quality of the figures in the merged PDF is not sufficient for the quality of this journal.

Consequently, I believe that the manuscript partially meets the journal's criteria of quality and relevance but it should clarify first all the questions above mentioned.

We greatly appreciate the editor's and reviewers' insightful and helpful suggestions concerning our manuscript entitled "An abscisic acid-induced transcription factor, *PsMYB306*, negatively regulates tree peony bud dormancy release" (PP2023-RA-01229). We took them seriously and made careful modifications to improve the quality of our manuscript. The detailed point-by-point responses to all the comments are listed below:

**Editor's comments:**

**Comment 1:**

*Please, consider describing in detail the plant samples used in all experiments, including the samples collected for RNASeq. Additionally, it is essential to clarify the experiments carried out by semi-quantitative RT-PCR and RT-qPCR, as well as normalizing expression using appropriate reference genes, instead of rRNA.*

**Response:**

We greatly appreciate the Editor's helpful comments. According to the suggestions, we added the detailed description about the plant samples used in many experiments, including plant materials and growth conditions, detection of endogenous hormones, RNA sequencing and data processing, subcellular localization and transactivation assay, generation of transgenic petunia plants, virus-induced gene silencing assay, measurement of chlorophyll content, and dual luciferase assay. The added statement or revision can be found in the sections of Materials and methods and Figure legends of our resubmitted manuscript (Line 568-570, 574, 577-578, 580-584, 587-589, 600-601, 604-606, 633, 637-638, 663-672, 683-689, 710-711, 717-718, 731-733).

For semi-quantitative RT-PCR and RT-qPCR, we need to clarify that all relative expression analysis of genes in the Figures were performed using RT-qPCR method. We only used an integrated semi-quantitative RT-PCR and RT-qPCR approach to examine the TRV accumulation levels in systemically-infected tree peony leaves with TRV constructs, which were shown in Supplemental Figure S3 and S5. The purpose of using both methods is to better indicate the successful replication and movement of TRV constructs in tree peony plants, thus supporting the effectiveness of TRV-based VIGS system in this plant species. We sincerely apologize for the unclear statement about the use of semi-quantitative RT-PCR and RT-qPCR methods. We changed 'Relative expression levels' to 'Quantitative real-time PCR analysis of expression levels' in Figure legends. Based on the comment from Reviewer #1, we agree that RT-qPCR is effective enough to check gene expression levels or TRV

accumulation levels, and the semi-quantitative method is unacceptable in the current day. Therefore, we deleted the semi-quantitative RT-PCR results in Supplemental Figures (S3 and S5) and the corresponding statement in Materials and methods (Line 278, 343, 646, 649).

For reference genes, we used *18S rRNA* and *26S rRNA* to normalize gene expression levels in tree peony and petunia, respectively. Although the two *rRNA*s are quite abundant in the samples, they are commonly used as internal controls since their expression is independent of developmental stages and external stimuli in many plant species. *18S rRNA* has been identified as the most stable reference gene in eggplant (Gantasala et al., 2013), tea plant (Wang et al., 2017), seashore mallow (Tang et al., 2015), and some cereals (Jarošová and Kundu, 2010). It has also been used for gene expression analysis in tree peony plants (Guo et al., 2018; Mao et al., 2022). Moreover, *26S rRNA* as PCR standard for determining the expression levels of genes has been widely used (Singh et al., 2004). We have performed functional analysis of a number of genes in petunia, and routinely used the *26S rRNA* as the reference gene (Yin et al., 2015; Sun et al., 2017; Wang et al., 2018; Ji et al., 2023). Even so, we followed the Editor's kind suggestions to find more appropriate reference genes. In some reports, *PsActin* was used as an internal control in gene expression analysis associated with bud dormancy release in tree peony (Zhang et al., 2020; Gao et al., 2023). *PhEF1α* was identified as the optimal reference gene during leaf and flower development in petunia (Mallona et al., 2010). Therefore, we used *PsActin* and *PhEF1α* as new reference genes in tree peony and petunia, respectively, to re-perform gene expression analysis based on RT-qPCR. The results showed that the new data displayed very similar expression patterns as the previous data. We added the new expression data in the resubmitted manuscript (Figure 4, 5, 6, 7, and 9; Supplemental Figure S3, S5, and S6). The relevant modification was made in the section of Materials and methods and Figure legends. Two relevant references for the use of *PsActin* (Gao et al., 2023) and *PhEF1α* (Mallona et al., 2010) were also added (Line 651-652).

**References:**

**Gantasala NP, Papolu PK, Thakur PK, Kamaraju D, Sreevathsa R, Rao U** (2013) Selection and validation of reference genes for quantitative gene expression studies by real-time PCR in eggplant (*Solanum melongena* L). BMC Res Notes **6:** 312

**Gao L, Niu D, Chi T, Yuan Y, Liu C, Gai S, Zhang Y** (2023) PsRGL1 negatively regulates chilling-and gibberellin-induced dormancy release by PsF-box1-mediated targeting for proteolytic degradation in tree peony. Hort Res **10:** uhad044

**Guo L, Guo D, Yin W, Hou X** (2018) Tolerance strategies revealed in tree peony (*Paeonia suffruticosa*; Paeoniaceae) ecotypes differentially adapted to desiccation. Appl Plant Sci **6:** e01191

**Jarošová J, Kundu JK** (2010) Validation of reference genes as internal control for studying viral infections in cereals by quantitative real-time RT-PCR. BMC Plant Biol **10:** 146

**Ji X, Xin Z, Yuan Y, Wang M, Lu X, Li J, Zhang Y, Niu L, Jiang C, Sun, D** (2023) A petunia transcription factor, *PhOBF1*, regulates flower senescence by modulating gibberellin biosynthesis. Hort Res **10:** uhad022

**Mallona I, Lischewski S, Weiss J, Hause B, Egea-Cortines M** (2010) Validation of reference genes for quantitative real-time PCR during leaf and flower development in *Petunia hybrida*. BMC Plant Biol **10:** 4.

**Mao Y, Ji X, Meng Q, Xu Z, Yuan Y, Li M, Niu L, Zhang Y, Sun D** (2022) Contribution of anthocyanin and polyunsaturated fatty acid biosynthesis to cold tolerance during bud sprouting in tree peony. Ind Crop Prod **188:** 115563

**Singh K, Raizada J, Bhardwaj P, Ghawana S, Rani A, Singh H, Kaul K, Kumar S** (2004) *26S rRNA*-based internal control gene primer pair for reverse transcription-polymerase chain reaction-based quantitative expression studies in diverse plant species. Anal Biochem **335:** 330-333

**Sun D, Li S, Niu L, Reid MS, Zhang Y, Jiang CZ** (2017) *PhOBF1*, a petunia ocs element binding factor, plays an important role in antiviral RNA silencing. J Exp Bot **68:** 915-930

**Tang XL, Wang HY, Shao CY, Shao HB** (2015) Reference gene selection for qPCR normalization of *Kosteletzkya virginica* under salt stress. BioMed Res Int **823806:** 1-8

**Wang H, Chang X, Lin J, Chang Y, Chen JC, Reid MS, Jiang CZ** (2018) Transcriptome profiling reveals regulatory mechanisms underlying corolla senescence in petunia. Hort Res **5:** 16

**Wang ML, Li QH, Xin HH, Chen X, Zhu XJ, Li XH** (2017) Reliable reference genes for normalization of gene expression data in tea plants (*Camellia sinensis*) exposed to metal stresses. PLoS ONE **12:** e0175863

**Yin J, Chang X, Kasuga T, Bui M, Reid MS, Jiang CZ** (2015) A basic helix-loop-helix transcription factor, PhFBH4, regulates flower senescence by modulating ethylene biosynthesis pathway in petunia. Hort Res **2:** 15059

**Zhang T, Yuan Y, Zhan Y, Cao X, Liu C, Zhang Y, Gai S** (2020). Metabolomics analysis reveals Embden Meyerhof Parnas pathway activation and flavonoids accumulation during dormancy transition in tree peony. BMC Plant Biol **20:** 484

**Reviewer #1's comments:**

**Comment 1:**

*I have many comments and suggestions in the marked up manuscript. Overall, the concept was interesting and based on reasonable data. I understand that the authors want to hold on to the idea of an advancement in the field, but really, we have known for a long time that manipulating ABA and GA levels will effect bud dormancy. We have known also for quite some time that related members of the MYB family alter ABA levels by binding to the promoters of and enhancing expression of NCEDs and thus increasing ABA production. Thus, the results, although novel for tree peony, are not particularly surprising. That said, this is a solid bit of work. It does add to the base of knowledge on dormancy by confirming the solid hypothesis that MYB306 is differentially expressed (down-regulated) during endodormancy breaking, and plays a direct role in reducing the expression of an NCED known to be involved in ABA level maintenance.*

**Response:**

We thank the reviewer for the insightful comments. We fully agree that ABA and GAs are two critical phytohormones during the regulation of bud dormancy under low temperature. Many studies have revealed that the treatments with ABA or GAs will inhibit or promote bud dormancy release, respectively (Li et al., 2018; Zhang et al., 2021). However, most studies focused on the variation of two hormone contents and associated gene expression levels during bud dormancy transition, or the impact of exogenous applications of two hormones on the dormancy release. Little is known about the transcriptional regulatory mechanisms underlying the crosstalk between ABA and GAs in the buds. Only a few reports suggested the regulatory roles of transcription factors, such as SVP-like (SVL) in poplar (Singh et al., 2018) and PpyABF3 in pear (Yang et al., 2023), in bud dormancy release by integrating both ABA and GA signals. In particular, the DAM/SVPs, belonging to MADS-box transcription factor family, are considered central regulators during bud dormancy release (Falavigna et al., 2019). We believe that our data provided novel insights into the crucial involvement of MYB transcription factors in cold-induced bud dormancy release by regulating the antagonism of ABA to GAs. It is definitely true that a few members of the MYB family affect ABA levels by specifically regulating the key ABA biosynthetic genes *NCED*s, such as Myb10-D in wheat (Lang et al., 2021), SsMYB113 in schima (Zhang et al., 2022), and GhMYB102 in cotton (Liu et al., 2023). Compared to PsMYB306 (subgroup 1), these MYBs seem to be the

members of different subgroups (5, 6, and 11, respectively) according to the classification of Arabidopsis MYB family (Stracke et al., 2001). They have been identified as the regulators of seed development or drought stress tolerance by altering ABA content. Whether these MYBs are involved in the regulation of ABA- and GA-mediated bud dormancy remain largely unknown. This further supports the novel role of PsMYB306 in bud dormancy regulation from the perspective of MYB family.

It is worth mentioning that the plant species tree peony, as one of the most famous traditional flowers in China, has extremely high ornamental significance. In recent years, the great medicinal and oil values of tree peony roots and seeds, respectively, have also been found and investigated (Yan et al., 2021; Deng et al., 2022). In the huge tree peony industry, the off-season cultivation occupies a large part to ensure the continuous production of floral products. Therefore, the understanding of molecular regulatory mechanisms underlying tree peony bud dormancy release is quite essential, which has attracted increased attention of some researchers in the floriculture field. Our studies here could provide a theoretical framework to manipulate the bud dormancy release through a MYB gene-specific genetic engineering of tree peony plants. We believe that this is of great significance for improving the application of tree peony products and further accelerating the whole industry development in the future.

**References:**

**Deng R, Gao J, Yi J, Liu P** (2022) Could peony seeds oil become a high-quality edible vegetable oil? The nutritional and phytochemistry profiles, extraction, health benefits, safety and value-added-products. Food Res Int **156:** 111200

**Falavigna VDS, Guitton B, Costes E, Andrés F** (2019) I want to (bud) break free: the potential role of DAM and SVP-like genes in regulating dormancy cycle in temperate fruit trees. Front Plant Sci **9:** 1990

**Lang J, Fu Y, Zhou Y, Cheng M, Deng M, Li M, Zhu T, Yang J, Guo X, Gui L, Li L, Chen Z, Yi Y, Zhang L, Hao M, Huang L, Tan C, Chen G, Jiang Q, Qi P, Pu Z, Ma J, Liu Z, Liu Y, Luo M, Wei Y, Zheng Y, Wu Y, Liu D, Wang J** (2021) *Myb10-D* confers *PHS-3D* resistance to pre-harvest sprouting by regulating *NCED* in ABA biosynthesis pathway of wheat. New Phytol **230:** 1940-1952

**Li J, Xu Y, Niu Q, He L, Teng Y, Bai S** (2018) Abscisic acid (ABA) promotes the induction and maintenance of pear (*Pyrus pyrifolia* white pear group) flower bud endodormancy. Int J Mol Sci **19:** 310

**Liu R, Shen Y, Wang M, Liu R, Cui Z, Li P, Wu Q, Shen Q, Chen J, Zhang S, Liu S, Ma H, Pang C, Ge C** (2023) GhMYB102 promotes drought resistance by regulating drought-responsive genes and ABA biosynthesis in cotton (*Gossypium hirsutum* L.). Plant Sci **329:** 111608

**Singh RK, Maurya JP, Azeez A, Miskolczi P, Tylewicz S, Stojkovic K, Delhomme N, Busov V, Bhalerao RP** (2018). A genetic network mediating the control of bud break in hybrid aspen. Nat Commun **9:** 4173

**Stracke R, Werber M, Weisshaar B** (2001) The R2R3-MYB gene family in *Arabidopsis thaliana*. Curr Opin Plant Biol **4:** 447-456

**Yan Z, Xie L, Li M, Yuan M, Tian Y, Sun D, Zhang Y, Niu L** (2021) Phytochemical components and bioactivities of novel medicinal food-peony roots. Food Res Int **140:** 109902

**Yang Q, Wu X, Gao Y, Ni J, Li J, Pei Z, Bai S, Teng Y** (2023) PpyABF3 recruits the COMPASS-like complex to regulate bud dormancy maintenance via integrating ABA signaling and GA catabolism. New Phytol **237:** 192-203

**Zhang G, Yu Z, Yao B, Teixeira da Silva JA, Wen D** (2022) SsMYB113, a *Schima superba* MYB transcription factor, regulates the accumulation of flavonoids and functions in drought stress tolerance by modulating ROS generation. Plant Soil **478:** 427-444

**Zhang Y, Yuan Y, Liu Z, Zhang T, Li F, Liu C, Cai S** (2021) $GA_3$ is superior to $GA_4$ in promoting bud endodormancy release in tree peony (*Paeonia suffruticosa*) and their potential working mechanism. BMC Plant Biol **21:** 323

**Comment 2:**

*There is one significant criticism though, and that was the use of semi-quantitative PCR to examine the expression of multiple genes. This is unacceptable in the current day in age where qRT-PCR is available. Unless data is provided that shows that the PCR reactions were stopped for analysis during the logarithmic stage, the data is completely unreliable. Thus any data that used this technique needs to be reassessed using appropriate methods. As some of this data (as best I can tell from the methods section and the figure legends) are key to the results and discussion, several of the key "findings" are not publishable. I understand the expense of doing so many qRT-PCR runs, but it is unavoidable. One option would be to collect samples for analysis at various cycles and demonstrate that the results are in the logarithmic phase for both the test gene and the internal control.*

**Response:**

Thanks for the reviewer's helpful suggestions. We need to clarify that all data of gene

expression analysis in the Figures of our manuscript were from quantitative real-time PCR (qRT-PCR) assay. We only used a combined method of semi-quantitative RT-PCR and qRT-PCR to detect the accumulation levels of TRV RNAs (RNA1 and RNA2) in systemically-infected tree peony leaves with TRV constructs, which were shown in Supplemental Figure S3 and S5. The reason we used both methods is to better demonstrate the efficient virus propagation and movement in tree peony buds or leaves, thereby suggesting a successful application of TRV-based VIGS system in tree peony plants. We feel very sorry for the unclear statement about the expression assessment methods in the sections of Materials and methods and Figure legends. Accordingly, we clearly described 'Relative expression levels' as 'Quantitative real-time PCR analysis of expression levels' in Figure legends of our resubmitted manuscript. In addition, we fully agree that the semi-quantitative RT-PCR method is unacceptable in the current day in age where qRT-PCR is available. It is very difficult to determine if the analysis results at a certain PCR cycle are in the logarithmic phase. Therefore, we removed the semi-quantitative RT-PCR results in Supplemental Figure S3 and S5 (Line 278, 343). The corresponding statement about the use of semi-quantitative RT-PCR method was also deleted in Materials and methods (Line 646, 649).

**The comments in the marked up manuscript:**

**Comment 3:**

*Line 27-28: So this is the switch from endodormancy to ecodormancy or is it the switch from ecodormancy to growth? If it is from ecodormancy, were they maintained in the cold???? I ask this because your last sentence in the abstract confused me and made me look back here.*

**Response:**

We greatly appreciate the reviewer's kind comments. The prolonged chilling treatment represents a cold stress at 2 ºC of up to 30 days. This chilling treatment began at the beginning of November, when the bud endo-dormancy of tree peony plants was initially established. It has been confirmed by some previous studies on tree peony (Huang et al., 2008; Zhang et al., 2015). In addition, the requirement of the chilling duration period varies among cultivars. For example, 0-4 ºC of chilling treatment for 21 days is required to break bud endo-dormancy of tree peony 'Luhehong' cultivar (Gao et al., 2023). Based on our practical studies, 2 ºC for 30 days is a proper treatment to completely break bud endo-dormancy of tree peony cultivar 'Yulouchun'. So the whole chilling treatment process is the switch from endo-dormancy to eco-dormancy. The bud growth did not occur at the later chilling stage due

to the repression of relatively low temperature (2 ºC). To clarify this switch, we made a relevant modification in the section of Abstract (Line 28, 30, 44). We also added a detailed description about the tree peony plants used for chilling treatment in Materials and methods (Line 568-570).

**References:**

**Gao L, Niu D, Chi T, Yuan Y, Liu C, Gai S, Zhang Y** (2023) PsRGL1 negatively regulates chilling-and gibberellin-induced dormancy release by PsF-box1-mediated targeting for proteolytic degradation in tree peony. Hort Res **10:** uhad044

**Huang X, Zhu W, Dai S, Gai S, Zheng G, Zheng C** (2008) The involvement of mitochondrial phosphate transporter in accelerating bud dormancy release during chilling treatment of tree peony (*Paeonia suffruticosa*). Planta **228:** 545-552

**Zhang Y, Zhang L, Gai S, Liu C, Lu S** (2015) Cloning and expression analysis of the *R2R3-PsMYB1* gene associated with bud dormancy during chilling treatment in the tree peony (*Paeonia suffruticosa*). Plant Growth Regul **75:** 667-676

**Comment 4:**

*Line 42: This was already stated above.*

**Response:**

Thanks for the reviewer's comment. It has been revealed that the members of NCED family probably play distinct roles in ABA biosynthesis or plant development (Fei et al., 2023; Liu et al., 2023). In our studies, various *NCED* isoforms with similar genetic structures have been found during the transcriptome data of tree peony in Supplemental Table S2 and S3. Although *PsMYB306* was found to be positively correlated with *PsNCED3* at the transcription level and PsMYB306 specifically bound to the promoter of *PsNCED3*, whether *PsNCED3* is involved in the regulation of ABA-mediated bud dormancy is still unknown. Thus, we used TRV-based VIGS method to further investigate the function of *PsNCED3*, and validated its important role in ABA production and bud dormancy regulation (Figure 9A-E). Based on the reasons above, we retained the statement about 'Silencing of *PsNCED3* also resulted in promoted bud break and growth' (Line 42-43).

**References:**

**Fei R, Guan S, Duan S, Ge J, Sun T, Sun X** (2023) Elucidating biological functions of *9-cis-epoxycarotenoid dioxygenase* genes involved in seed dormancy in *Paeonia lactiflora*.

Plants **12:** 710
**Liu J, Yuan X, Quan S, Zhang M, Kang C, Guo C, Zhang Z, Niu J** (2023) Genome-wide identification and expression analysis of *NCED* gene family in pear and its response to exogenous gibberellin and paclobutrazol. Int J Mol Sci **24:** 7566

**Comment 5:**

*Line 43-44: I am not sure it regulates the antagonism of GAs and ABAs, but rather just the levels of these hormones that act antagonistically.*

**Response:**

We thank the reviewer for the helpful comment. The antagonism of ABA and GAs during bud dormancy regulation has been widely revealed in many woody perennial plant species (Cooke et al., 2012). In tree peony, it has been reported that GA ($GA_3$ and $GA_4$) treatment promoted bud dormancy release, and decreased endogenous ABA content and expression levels of several ABA receptor genes (*PsPYL1*, *PsPYL8*, and *PsPYL12*) (Zhang et al., 2021). Similarly, our data in the VIGS assays showed that ABA treatment remarkably inhibited the production of GAs ($GA_1$ and $GA_3$) in TRV-*PsMYB306*-infected tree peony buds compared to the untreated ones, resulting in a slowed bud break and growth (Figure 6 and 7). We agree with the reviewer that the antagonism of ABA and GAs regulated by PsMYB306 is mainly manifested in the levels of these hormones. The strong evidence here revealed the direct involvement of PsMYB306 in the ABA biosynthesis. Thus, we changed 'regulating the antagonism of ABA to GAs' to 'regulating the ABA production' in Abstract (Line 45).

**References:**

**Cooke JE, Eriksson ME, Junttila O** (2012) The dynamic nature of bud dormancy in trees: environmental control and molecular mechanisms. Plant Cell Environ **35:** 1707-1728
**Zhang Y, Yuan Y, Liu Z, Zhang T, Li F, Liu C, Cai S** (2021) $GA_3$ is superior to $GA_4$ in promoting bud endodormancy release in tree peony (*Paeonia suffruticosa*) and their potential working mechanism. BMC Plant Biol **21:** 323

**Comment 6:**

*Line 50-52: ecodormancy does not improve bud sprouting. The authors made a shift from endo to eco dormancy here but did not introduce the concept. This needs to be rewritten to make more sense.*

**Response:**

We feel sorry for the inappropriate statement. As suggested by the reviewer, we re-wrote this part to make it more sense by changing 'It is also a crucial step to improve the quality of bud sprouting, plant growth, and flowering after winter' to 'It is also a crucial developmental step that affects bud sprouting, plant growth, and flowering'. Moreover, the revised statement was moved to the end of this paragraph (Line 58-59).

**Comment 7:**

*Line 54: This is also not entirely as true statement as endodormancy induction by temperature is species specific. Poplar for example, relies on day length alone for endodormancy induction.*

**Response:**

Thanks for the insightful suggestion. It is absolutely true that the induction of endo-dormancy for different plant species replies on various environmental signals, such as low temperature and short-day length. Therefore, we changed 'and low temperature is one of the main signals that induce growth arrest' to 'Low temperature and short-day length are two main signals that induce endo-dormancy and growth arrest', and also changed 'Meanwhile' to 'In many temperature-sensitive plants'. A proper reference (Yang et al., 2021) was also added (Line 53-55).

**References:**

**Yang Q, Gao Y, Wu X, Moriguchi T, Bai S, Teng Y** (2021) Bud endodormancy in deciduous fruit trees: Advances and prospects. Hort Res **8:** 139

**Comment 8:**

*Line 57: maybe move the Yamane reference statement here.*

**Response:**

We agree that, and moved the revised Yamane reference statement to the end of this paragraph (Line 58-59).

**Comment 9:**

*Line 72: Be specific as to the type of dormancy as they do differ.*

**Response:**

Sure. We clearly re-described 'during dormancy induction' as 'during endo-dormancy induction' (Line 74).

**Comment 10:**

*Line 78: what is meant by "meristem activation"? Is this the switch to ecodormancy?*

**Response:**

According to the given context in the reference (Zheng et al., 2018), the phrase ‘meristem activation’ means the endo-dormancy release of bud meristem, thereby representing the switch from endo-dormancy to eco-dormancy. We apologize for the confusing statement, and replaced ‘the GA-promoting effect on primordial growth and bud break was inhibited after meristem activation’ with ‘GAs inhibited bud endo-dormancy release and promoted bud primordia growth after dormancy release in grapevine’ (Line 79-80).

**Comment 11:**

*Line 80: endodormancy release?*

**Response:**

Correct. The phrase ‘bud meristem activity’ indicates endo-dormancy release. To make a proper description, we changed ‘the dormancy was maintained by ABA-mediated repression of bud meristem activity’ to ‘the endo-dormancy was maintained by ABA-mediated repression of bud meristem activation’ (Line 81-82).

**Comment 12:**

*Line 81: endo or eco???*

**Response:**

The ‘dormancy’ here represents endo-dormancy. So we changed ‘dormancy release’ to ‘endo-dormancy release’ (Line 83).

**Comment 13:**

*Line 82: what is "bud promotion"? endo or ecodormancy release????*

**Response:**

The ‘bud promotion’ here suggests endo-dormancy release. We are sorry for the inappropriate statement, and therefore replaced ‘bud promotion’ with ‘bud endo-dormancy release’. Meanwhile, the ‘dormancy release’ at the end of this sentence was changed to ‘bud break’ to avoid a repetitive description (Line 84-86).

**Comment 14:**

*Line 84-85: Oh, I think it is pretty well understood. Rinne's work pretty much nailed it in regards to endodormancy. Many other physiological studies have pretty much covered the mechanisms of the cross-talk through DELLAs and NCEDs.*

**Response:**

We thank the reviewer for pointing out the inappropriate statement, and fully agree that the crosstalk between ABA and GAs during bud dormancy release is well understood with many studies supporting this conclusion. Thus, we removed the previous sentence, and added a statement including a reference from Rinne's group as 'Although the crosstalk between ABA and GAs is well-known (Veerabagu et al., 2023), their roles in the regulation of bud endo-dormancy has not been thoroughly elucidated' (Line 86-88).

**References:**

**Veerabagu M, van der Schoot C, Turečková V, Tarkowská D, Strnad M, Rinne PL** (2023) Light on perenniality: Para-dormancy is based on ABA-GA antagonism and endo-dormancy on the shutdown of GA biosynthesis. Plant Cell Environ **46:** 1785-1804

**Comment 15:**

*Line 94: Start a new paragraph here.*

**Response:**

Sure. As suggested by the reviewer, we started a new paragraph here (Line 99).

**Comment 16:**

*Line 106-107: move this sentence to a new paragraph that explains why bud dormancy is important in tree peony- either at the beginning of the introduction or the end.*

**Response:**

Thanks for the helpful advice. The importance of bud dormancy for tree peony is reflected in the aspect of forcing culture. Forcing culture in winter is an important part of tree peony industry to extend the supply of floral products. Bud dormancy is the major constraining factor for the achievement of forcing culture. Therefore, the elucidation of molecular basis of bud dormancy is critical for tree peony. Our data presented here provided valuable insights into the genetic basis of bud dormancy, and would benefit future improvement of forcing culture in tree peony by breeding or bioengineering approach. We deleted the previous statement 'The off-season flowering will largely improve the commercial application of tree peonies'. An explanation why bud dormancy is important in tree peony was added in the

same paragraph and at the end of the Introduction (Line 111-114, 139-141).

**Comment 17:**

*Line 111: need a reference here.*

**Response:**

According to the reviewer's suggestion, we added a reference (Gai et al., 2013) here (Line 118).

**References:**

**Gai S, Zhang Y, Liu C, Zhang Y, Zheng G** (2013) Transcript profiling of *Paoenia ostii* during artificial chilling induced dormancy release identifies activation of GA pathway and carbohydrate metabolism. PLoS One **8:** e55297

**Comment 18:**

*Line 127: peony???*

**Response:**

The word 'petunia' here is correct, and it should not be 'peony'. Due to the recalcitrance of tree peony plants to genetic transformation, we used TRV-based VIGS assay in tree peony and ectopic overexpression assay in petunia to investigate the function of *PsMYB306*. This sentence is a brief description about why petunia was used as a model plant for ectopic overexpression assay in our work. To avoid the confusion, the previous sentence was re-described as 'petunia has been widely employed as a model platform for gene overexpression assays in floral crops' (Line 134-135).

**Comment 19:**

*Line 166: I am confused by this statement. Isn't stage one time = 0? If these genes are down regulated, what is that in comparison to?*

**Response:**

We are thankful for the kind comment, and agree that the statement here is inappropriate. The down-regulation represents the expression profiles of these genes from S1 (0 d after chilling treatment) to S3 (30 d after chilling treatment). That said, this is the gene expression trend at S2 and S3 in comparison to S1. To avoid the confusion, we changed 'at three stages of chilling stress' to 'from S1 to S3 under chilling stress' (Line 177). A similar revision was also made in the next paragraph (Line 200).

**Comment 20:**

*Line 174-175: Just correlation that does not contradict the established dogma. Not sure it "strongly supports"...*

**Response:**

We agree with the reviewer's comment, and removed the inappropriate statement (Line 192).

**Comment 21:**

*Line 183: see note above.*

**Response:**

As responded to Comment 19 above, we changed 'at three stages of chilling stress' to 'from S1 to S3 under chilling stress' (Line 200).

**Comment 22:**

*Line 283: Do the authors mean "restored growth inhibition"?*

**Response:**

We feel sorry for the inappropriate statement. The phrase 'restored the accelerated growth in *PsMYB306*-silenced buds' means 'reduced the growth of *PsMYB306*-silenced buds'. We made a relevant modification to make it clear to understand (Line 301).

**Comment 23:**

*Line 283-284: I think the authors do not want to say this as it does not match what was observed. And I'll ask here too. If ABA reduces growth in TRV-MYB306 plants, why doesn't it reduce growth in the empty vector control??? This requires an explanation.*

**Response:**

We thank the reviewer for the valuable comment. We agree that the statement here is incorrect. What we would like to express is that the treatment with ABA repressed the accelerated growth of *PsMYB306*-silenced buds, resulting in a similar growth phenotype between *PsMYB306*-silenced and empty vector-infected (non-silenced) buds. To make an correct statement, we changed 'the treatment with ABA restored the accelerated growth in *PsMYB306*-silenced buds to normal levels in non-silenced ones' to 'the treatment with ABA reduced the growth of *PsMYB306*-silenced buds, which was similar to that of empty vector-infected buds' (Line 301-302).

Regarding the impact of ABA treatment on the empty vector control, ABA application

actually reduced the growth of empty vector-infected buds when comparing the untreated ones in Figure 6. In detail, the empty vector-infected buds treated with ABA displayed bud break rates of 59.4% and 79.6% and plant heights of 3.9 cm and 5.9 cm at 7 and 14 days after infiltration, respectively (Figure 7B, C). In contrast, the untreated empty vector-infected buds showed bud break rates of 68.3% and 83.6% and plant heights of 4.3 cm and 6.7 cm at 7 and 14 days after infiltration, respectively (Figure 6C, D). The results indicate that ABA treatment also inhibited the bud break and growth of empty vector controls. A similar phenotype between *PsMYB306*-silenced and non-silenced buds after ABA treatment demonstrates that the decreased ABA production is the key factor for accelerated growth of *PsMYB306*-silenced buds, thereby supporting the pivotal role of PsMYB306 in the regulation of bud dormancy release and break by modulating the ABA content.

**Comment 24:**

*Line 320: Cool! I haven't seen one of these assays published in a long time. Old-school for the win! :-)*

**Response:**

We thank the reviewer for the kind comment very much. It is definitely true that these assays used for binding test are sort of old, but they are really effective methods to examine the transactivation of gene promoters by transcription factors for a long time.

**Comment 25:**

*Line 383: define this abbreviation.*

**Response:**

Thanks for the helpful advice. The full name of 'WER' is 'WEREWOLF', which was added where it firstly appeared (Line 232).

**Comment 26:**

*Line 430: Well yes, in as much as it alters the ABA to GA ratio and therefore inhibits GA-induced growth and likely prevents opening of the plasmodesmata required for dormancy breaking.*

**Response:**

Thanks. It is absolutely correct that plasmodesmata closure is associated with dormancy establishment by blocking the supply of growth-promoting signals to bud meristems, which is considered an ABA-dependent process (Tylewicz et al., 2018). Besides the inhibition of

GA-induced growth, ABA probably suppresses bud dormancy release by preventing the opening of plasmodesmata. It has been reported that the up-regulation of GA biosynthesis occurred concomitantly with the opening of plasmodesmata during dormancy release (Maurya and Bhalerao, 2017). The regulatory role of PsMYB306 in ABA-mediated plasmodesmata closure should be investigated in future work. We added a relevant description about the association of plasmodesmata with bud dormancy in Discussion (Line 465-472), and two new references were added.

**References:**


**Maurya JP, Bhalerao RP** (2017) Photoperiod-and temperature-mediated control of growth cessation and dormancy in trees: a molecular perspective. Ann Bot **120:** 351-360

**Tylewicz S, Petterle A, Marttila S, Miskolczi P, Azeez A, Singh RK, Immanen J, Mähler N, Hvidsten TR, Eklund DM, Bowman JL, Helariutta Y, Bhalerao RP** (2018) Photoperiodic control of seasonal growth is mediated by ABA acting on cell-cell communication. Science **360:** 212-215


**Comment 27:**

*Line 467-469: Really, it just increased the level of ABA. Thus it doesn't "regulate the antagonism" it really just alters the balance and the antagonism acts accordingly.*

**Response:**

We apologize for the inappropriate statement. According to the reviewer’s suggestion, we changed the previous statement ‘we determined the PsMYB306-regulated antagonism between ABA and GAs during bud dormancy release of tree peony in the present study’ to ‘we determined that PsMYB306 affected the ABA/GAs balance during bud dormancy release of tree peony in the present study’ (Line 497).

**Comment 28:**

*Line 483-484: You can bet that if ABA plays a role in leaf senescence, then MYB306 will also play a role... so long as it is induced/suppressed in the appropriate cells.*

**Response:**

Many pieces of evidence have suggested the crucial role of ABA in the regulation of leaf senescence (Takasaki et al., 2015; Mao et al., 2017; Fan et al., 2020; Wen et al., 2023). Based on the findings here, it is thus quite likely that PsMYB306 contributes to the modulation of leaf senescence. In subsequent studies, we will use a transient TRV-based VIGS system in

tree peony leaves or genetic transformation in model plant petunia to test this hypothesis.

**References:**

**Fan ZQ, Tan XL, Shan W, Kuang JF, Lu WJ, Lin HT, Su XG, Lakshmanan P, Zhao ML, Chen JY** (2020) Involvement of BrNAC041 in ABA-GA antagonism in the leaf senescence of Chinese flowering cabbage. Postharvest Biol Tec **168:** 111254

**Mao C, Lu S, Lv B, Zhang B, Shen J, He J, Luo L, Xi D, Chen X, Ming F** (2017) A rice NAC transcription factor promotes leaf senescence via ABA biosynthesis. Plant Physiol **174:**1747-1763

**Takasaki H, Maruyama K, Takahashi F, Fujita M, Yoshida T, Nakashima K, Myouga F, Toyooka K, Yamaguchi-Shinozaki K, Shinozaki, K** (2015) SNA-As, stress-responsive NAC transcription factors, mediate ABA-inducible leaf senescence. Plant J **84:** 1114-1123

**Wen B, Zhao X, Gong X, Zhao W, Sun M, Chen X, Li D, Li L, Xiao W** (2023) The NAC transcription factor MdNAC4 positively regulates nitrogen deficiency-induced leaf senescence by enhancing ABA biosynthesis in apple. Mol Hortic **3:** 5

**Comment 29:**

*Line 514: endo or ecodormancy?*

**Response:**

Here is endo-dormancy. We changed ‘bud dormancy’ to ‘bud endo-dormancy’ to make a clear statement (Line 543).

**Comment 30:**

*Line 532: The authors need to add some discussion of the previous studies that also demonstrated that this gene (or likely orthologues of this gene) were shown to directly bind to NCEDs and increase ABA levels-see for example Lee K, Seo PJ. Coordination of seed dormancy and germination processes by MYB96. Plant signaling & behavior. 2015 Sep 2;10(9):e1056423. and other follow-on studies.*

**Response:**

We are grateful to the reviewer for the insightful suggestion. We fully agree that some discussion of the previous studies about this gene or its orthologues should be added. Similar to PsMYB306, its homolog MYB96 was reported to activate ABA biosynthesis by binding to the promoters of *NCED2* and *NCED6*, thereby contributing to the modulation of Arabidopsis seed dormancy (Lee et al., 2015; Lee and Seo, 2015). Considering the similarity and

difference between ABA-mediated bud and seed dormancy (Wang et al., 2016), a complex regulatory network by PsMYB306 may exist in the dormancy process of various tree peony organs. We added this statement in an appropriate part of Discussion, and three new relevant references were also added (Line 440-445).

**References:**

**Lee HG, Lee K, Seo PJ** (2015) The Arabidopsis MYB96 transcription factor plays a role in seed dormancy. Plant Mol Biol **87:** 371-381

**Lee K, Seo PJ** (2015) Coordination of seed dormancy and germination processes by MYB96. Plant Signal Behav **10:** e1056423

**Wang D, Gao Z, Du P, Xiao W, Tan Q, Chen X, Li L, Gao D** (2016) Expression of ABA metabolism-related genes suggests similarities and differences between seed dormancy and bud dormancy of peach (*Prunus persica*). Front Plant Sci **6:** 1248

**Comment 31:**

*Line 538-539: how was dormancy confirmed?*

**Response:**

We thank for the reviewer's insightful comment. The tree peony plants were grown in the natural environment until the beginning of November, when the plant growth was already terminated and flower bud differentiation was completed. It was the time for tree peony buds entering the endo-dormancy state. Some previous studies supported the establishment of tree peony bud dormancy at this time point (Huang et al., 2008; Zhang et al., 2015). Moreover, early November is also a commonly selected time point to initiate the forcing culture of tree peony in winter. Thus, we chose early November for chilling treatment of the buds from tree peony cultivar 'Yulouchun'. We made a relevant modification to detail the establishment of bud endo-dormancy and added two references in Materials and methods (Line 568-570).

**References:**

**Huang X, Zhu W, Dai S, Gai S, Zheng G, Zheng C** (2008) The involvement of mitochondrial phosphate transporter in accelerating bud dormancy release during chilling treatment of tree peony (*Paeonia suffruticosa*). Planta **228:** 545-552

**Zhang Y, Zhang L, Gai S, Liu C, Lu S** (2015) Cloning and expression analysis of the *R2R3-PsMYB1* gene associated with bud dormancy during chilling treatment in the tree peony (*Paeonia suffruticosa*). Plant Growth Regul **75:** 667-676

**Comment 32:**

*Line 581-582: What mapping software was used for expression quantification? Also, note specifically the Fasta file that was used to map the reads to. Indeed, it might be useful to include that file as a supplemental file.*

**Response:**

The mapping software used for expression quantification is Cufflinks program (version 2.1.1) (Trapnell et al., 2010), which were added in the section of Materials and methods (Line 620). We agree that the Fasta file of unigene sequences used to map the reads is useful, and it is really a big file for the upload. The Fasta file was added as a new Supplemental Figure S7 (Line 617, 797-798).

**References:**

**Trapnell C, Roberts A, Goff L, Pertea G, Kim D, Kelley DR, Pimentel H, Salzberg SL, Rinn JL, Pachter L** (2012) Differential gene and transcript expression analysis of RNA-seq experiments with TopHat and Cufflinks. Nat Protoc **7:** 562-578

**Comment 33:**

*Line 609-610: Any analysis for which semi-quantitative PCR was used should be deleted from the manuscript or re-run using qRT-PCR methods. This method is not reliable.*

**Response:**

We are very thankful for the reviewer's valuable comment. As responded to Comment 2 above, we only used an integrated method of semi-quantitative RT-PCR and qRT-PCR to detect the TRV accumulation levels in systemically-infected tree peony leaves with TRV constructs, which were shown in Supplemental Figure S3 and S5. All the other expression analysis was performed using qRT-PCR methods. According to the reviewer's suggestion, we deleted all data of semi-quantitative PCR analysis and the corresponding statement about results and methods in the sections of Supplemental data, Results, and Materials and methods (Line 278, 343, 646, 649).

**Comment 34:**

*Line 614-615: Good that two genes were used, but the authors should provide evidence that these two genes are not themselves differentially expressed during dormancy transitions or ABA/GA treatments. The authors could use their RNAseq data as evidence, but they should*

*provide the relative abundance of any cDNAs for which the primer pairs would likely bind and amplify.*

**Response:**

We greatly appreciate the reviewer's helpful comment. In the present study, we used *18S rRNA* and *26S rRNA* as reference genes to normalize gene expression levels in tree peony and petunia, respectively. Of them, *18S rRNA* has been identified as the most stable reference gene in some plant species, including eggplant (Gantasala et al., 2013), tea plant (Wang et al., 2017), and some cereals (Jarošová and Kundu, 2010). It has also been used for gene expression analysis in tree peony leaves and buds (Guo et al., 2018; Mao et al., 2022). Additionally, *26S rRNA* as a reference gene to standardize gene expression levels has been widely used (Singh et al., 2004). We have used *26S rRNA* as an internal control for expression analysis of a number of genes in petunia leaves and flowers (Yin et al., 2015; Sun et al., 2017; Ji et al., 2023). Thus, a number of previous publications support the effectiveness of *18S rRNA* and *26S rRNA* used as reference genes, and that is the reason we selected them.

Based on the Editor's kind suggestion, the *rRNA*s seem not to be the optimal reference genes owing to their high abundances in various plant tissues. Therefore, we followed this suggestion to find more appropriate reference genes, which are *PsActin* in tree peony and *PhEF1α* in petunia. *PsActin* has been used as an internal control in bud dormancy-associated gene expression analysis in tree peony (Zhang et al., 2020; Gao et al., 2023). Notably, our RNA-Seq data showed that *PsActin* (ID: Unigene0012907) was not differentially expressed during chilling-induced bud dormancy release. Specifically, the mean RPKM values of *PsActin* at S1, S2, and S3 were 493.57, 487.75, and 489.43, respectively. Our list of differentially expressed unigenes included no *PsActin* or its any actin isoforms (Supplemental Table S3), further confirming the stable expression of *PsActin* during bud dormancy transition. *PhEF1α* was characterized as the optimal reference gene during leaf and flower development in petunia (Mallona et al., 2010). We believe that *PsActin* and *PhEF1α* are more appropriate reference genes than *rRNA*s. Next, we used *PsActin* and *PhEF1α* as new reference genes to re-perform qRT-PCR analysis instead of *18S rRNA* and *26S rRNA*. The results showed that the new expression data are very similar to the previous data.

Moreover, it is definitely true that the primer pairs would likely bind and amplify in the course of qRT-PCR assay. The optimal primer design is quite important for the accuracy of experimental data. In our assay, we used the same primer pairs as those which have been reported and validated in previous studies (Mallona et al., 2010; Gao et al., 2023). We further used Primer Premier 5.0 software to double-check the quality of primer pairs to avoid

potential hairpin and primer-dimer formation. This software was also used for primer design and quality evaluation of other gene primer pairs in our studies. Furthermore, No hairpin or primer-dimer was observed in the amplification products of both new reference genes when analyzing melting curves, which displayed a single high peak. This suggests that the primer pairs used here are quite effective to normalize gene expression levels. Based on the response above, we added the new expression data in the resubmitted manuscript (Figure 4, 5, 6, 7, and 9; Supplemental Figure S3, S5, and S6). The statement about Primer Premier 5.0 software and two references (Mallona et al., 2010; Gao et al., 2023) were also added. The relevant modification was made in the section of Materials and methods and Figure legends (Line 651-652, 654-655).

**References:**

**Gantasala NP, Papolu PK, Thakur PK, Kamaraju D, Sreevathsa R, Rao U** (2013) Selection and validation of reference genes for quantitative gene expression studies by real-time PCR in eggplant (*Solanum melongena* L). BMC Res Notes **6:** 312

**Gao L, Niu D, Chi T, Yuan Y, Liu C, Gai S, Zhang Y** (2023) PsRGL1 negatively regulates chilling-and gibberellin-induced dormancy release by PsF-box1-mediated targeting for proteolytic degradation in tree peony. Hort Res **10:** uhad044

**Guo L, Guo D, Yin W, Hou X** (2018) Tolerance strategies revealed in tree peony (*Paeonia suffruticosa*; Paeoniaceae) ecotypes differentially adapted to desiccation. Appl Plant Sci **6:** e01191

**Jarošová J, Kundu JK** (2010) Validation of reference genes as internal control for studying viral infections in cereals by quantitative real-time RT-PCR. BMC Plant Biol **10:** 146

**Ji X, Xin Z, Yuan Y, Wang M, Lu X, Li J, Zhang Y, Niu L, Jiang C, Sun, D** (2023) A petunia transcription factor, *PhOBF1*, regulates flower senescence by modulating gibberellin biosynthesis. Hort Res **10:** uhad022

**Mallona I, Lischewski S, Weiss J, Hause B, Egea-Cortines M** (2010) Validation of reference genes for quantitative real-time PCR during leaf and flower development in *Petunia hybrida*. BMC Plant Biol **10:** 4.

**Mao Y, Ji X, Meng Q, Xu Z, Yuan Y, Li M, Niu L, Zhang Y, Sun D** (2022) Contribution of anthocyanin and polyunsaturated fatty acid biosynthesis to cold tolerance during bud sprouting in tree peony. Ind Crop Prod **188:** 115563

**Singh K, Raizada J, Bhardwaj P, Ghawana S, Rani A, Singh H, Kaul K, Kumar S** (2004) *26S rRNA*-based internal control gene primer pair for reverse transcription-polymerase chain

reaction-based quantitative expression studies in diverse plant species. Anal Biochem **335:** 330-333

**Sun D, Li S, Niu L, Reid MS, Zhang Y, Jiang CZ** (2017) *PhOBF1*, a petunia ocs element binding factor, plays an important role in antiviral RNA silencing. J Exp Bot **68:** 915-930

**Wang ML, Li QH, Xin HH, Chen X, Zhu XJ, Li XH** (2017) Reliable reference genes for normalization of gene expression data in tea plants (*Camellia sinensis*) exposed to metal stresses. PLoS ONE **12:** e0175863

**Yin J, Chang X, Kasuga T, Bui M, Reid MS, Jiang CZ** (2015) A basic helix-loop-helix transcription factor, PhFBH4, regulates flower senescence by modulating ethylene biosynthesis pathway in petunia. Hort Res **2:** 15059

**Zhang T, Yuan Y, Zhan Y, Cao X, Liu C, Zhang Y, Gai S** (2020). Metabolomics analysis reveals Embden Meyerhof Parnas pathway activation and flavonoids accumulation during dormancy transition in tree peony. BMC Plant Biol **20:** 484

**Comment 35:**

*Figure 2: italics.*

**Response:**

Thanks. We changed 'italic types' to 'italics' as advised by the reviewer.

**Comment 36:**

*Figure 6: I think this is the semi-quantitative PCR. If so this all needs to be redone!!!*

**Response:**

As responded to Comment 2 above, all gene expression analysis in the Figures was performed by qRT-PCR. We only used both semi-quantitative RT-PCR and qRT-PCR methods to detect TRV accumulation levels, which were shown in Supplemental Figure S3 and S5. Therefore, this is the qRT-PCR analysis.

**Comment 37:**

*Figure 7: Need TRV and TRV-MYB306 untreated to make statement that ABA restored normal growth to TRV-MYB306 plants. I suppose one could look at figure 6 to see, but the difference in growth state, is not easy to determine looking between the two figures.*

**Response:**

Thanks for the reviewer's kind suggestion. I need to clarify that the apical buds at the same developmental levels were selected for VIGS and ABA treatment assays. To maintain a

uniform growth state, the TRV- and TRV-*PsMYB306*-infected buds treated with ABA and the untreated ones were grown under the same environmental conditions (a growth chamber at 20 ºC with a 16/8 h light/dark photoperiod). Particularly, the untreated TRV- and TRV-*PsMYB306*-infected buds have been shown in Figure 6, which could be used as a reliable comparison with the ABA-treated buds in terms of phenotypes and experimental data. It might be a repetitive display if the untreated buds were added in Figure 7 for this manuscript. It is noteworthy that our main focus is on the effect of ABA application on the dormancy release and growth of TRV-*PsMYB306*-infected buds, compared to the empty vector-infected ones. After ABA application, the accelerated growth of TRV-*PsMYB306*-infected buds was inhibited, which was similar to that of empty vector-infected ones. The bud break rate, plant height, GA contents, and gene expression levels supported the phenotypic similarity. These results suggest that PsMYB306-mediated ABA plays an essential role in bud dormancy release. Thus, ABA-treated empty vector-infected buds seem to be an effective enough and more important control than the untreated ones in the ABA treatment assay. In addition, we realize that the statement that ABA restored normal growth is inappropriate. So we changed 'the treatment with ABA restored the accelerated growth in *PsMYB306*-silenced buds to normal levels in non-silenced ones' to 'the treatment with ABA reduced the growth of *PsMYB306*-silenced buds, which was similar to that of empty vector-infected buds' in Results (Line 301-302).

**Comment 38:**

*Figure 7: Why doesn't ABA reduce growth in both treatments????*

**Response:**

We appreciate the reviewer's kind comment. As responded to Comment 23 above, apart from TRV-*PsMYB306*-infected buds, ABA treatment also reduced the growth of empty vector-infected buds compared to the untreated ones in Figure 6. For the specific data, the ABA-treated empty vector controls showed bud break rates of 59.4% and 79.6% and plant heights of 3.9 cm and 5.9 cm at 7 and 14 days after infiltration, respectively (Figure 7B, C). In contrast, the untreated controls showed bud break rates of 68.3% and 83.6% and plant heights of 4.3 cm and 6.7 cm at 7 and 14 days after infiltration, respectively (Figure 6C, D). This suggests that ABA treatment inhibited the bud break and growth of empty vector-infected buds to a certain extent.

**Additional response:**

We noticed that the reviewer made some direct modifications to incorrect or inappropriate words and phrases in our submitted manuscript without any comments. We are very grateful to the reviewer for those serious and careful revisions. We fully agree with all modifications made to the text by the reviewer, which were followed by us in the resubmitted manuscript (Line 35, 38-39, 51, 56, 74-75, 78, 93, 110, 118, 121, 125-126, 128, 169, 281, 291, 320, 505, 510, 537-538, 555).

**Reviewer #2's comments:**

**Comment 1:**

*In the Introduction and along the entire work, genes should be spell out to describe to the reader what pathways are described.*

**Response:**

We greatly appreciate the reviewer's valuable suggestion. We fully agree that the abbreviated gene names should be spelled out to give the readers a clear description. Thus, we added the full names of the abbreviated genes when they firstly appeared in the sections of Abstract, Introduction, Results, Discussion, and Materials and methods (Line 32-33, 39-40, 91-92, 94, 96-97, 122, 175-177, 179-182, 184-190, 202-203, 208, 232, 353, 457-458, 503, 505-506, 652).

**Comment 2:**

*In the Methodology and Results described: The RNAseq samples used where from the buds at 0(S1), 15 (S2) and 30 (S3) days after chilling treatment. Could you explain why? How are the morphology of these samples? Is there any picture about it? How much time does it take for a bud to release dormancy in natural conditions? Without the artificial chilling treatment? Is there a control where no chilling treatment was used? In my opinion, this should be the control of the whole experiment, not only the 0 chilling treatment, but the one in normal conditions to compare with the S2 and S3, for example.*

**Response:**

We thank the reviewer for the helpful comments. In the present study, we need to select a complete process of cold-induced bud dormancy release for a large-scale transcriptome analysis in tree peony. The objective is to screen a number of critical differentially expressed transcription factors which regulate bud dormancy transition under low temperature. It is well-known that low temperature is a key signal to promote bud dormancy release in tree

peony (Zhang et al., 2015; Zhang et al., 2020). However, the requirement of the chilling duration period varies among tree peony cultivars (Gao et al., 2023), which is dependent on the specific temperature and time span. Here, the chilling treatment began in early November, when the tree peony bud dormancy was initially established (Huang et al., 2008). According to our practical studies, 2 ºC for 30 days is a proper treatment to completely bread bud dormancy of tree peony cultivar 'Yulouchun'. Besides, our results showed a continuous reduction and elevation in the levels of ABA and bioactive GAs ($GA_1$ and $GA_3$) during chilling treatment (Figure 1B, C). Their accumulation levels tended to be stable at the later stages, further supporting that 30 days of chilling treatment at 2 ºC represent a complete process of cold-induced bud dormancy release. Given that 0, 15, and 30 days are important nodes in this release process, we therefore chose these three time points for RNA-Seq analysis. We added this explanation about the timing selection in the sections of Results and Materials and methods (Line 150-153, 568-569).

For the morphology of these samples, the tree peony buds did not show very significant phenotypic changes under chilling conditions. At the later stages of bud dormancy release, some minor alterations in bud morphology could be observed, including increased bud size, color change of outer scales, and slight opening of apical scales, which was similar to the phenotypes observed in chilling-treated buds from tree peony cultivar 'Luhehong' (Zhang et al., 2020). These minor morphological changes demonstrate that a complete dormancy release of tree peony buds is gradually induced by the chilling stress. Yes, we have the pictures about the bud morphology at three stages of chilling treatment. The bud phenotypes at 0 (S1) and 30 (S3) days have been already shown in Figure 1D. Thus, we added a new picture about the bud phenotype at 15 (S2) days.

In a natural environment without artificial chilling treatment, when the temperature continues to decrease between September and October, the tree peony buds will enter a stage of endo-dormancy in early November (Huang et al., 2008; Zhang et al., 2023). At this time, the plant growth was already terminated and flower bud differentiation was completed. After a prolonged cold stress (chilling or freezing), the bud endo-dormancy is released and shifted to another stage of eco-dormancy in January. Most buds will sprout in the next spring with a rise in ambient temperature. Therefore, it appears to take about two months for the buds to release endo-dormancy under natural conditions. The bud endo-dormancy release is the main focus in our studies here. We agree with the reviewer's point that a control with no chilling treatment would be useful in the whole experiment. However, the ambient temperature is always changing and quite unstable in a natural environment. Meanwhile, the tree peony buds

are easy to be affected by other environmental factors, such as day length, atmospheric humidity, wind, and insects. Thus, the use of no chilling treatment in normal conditions may cause some inconsistencies in the results. On the other hand, selecting a specific and stable temperature is a good way to simulate no chilling treatment, but what temperature is the most appropriate requires a further investigation about natural cold accumulation rates and others. Moreover, the bud growth almost ceases during cold-induced bud endo-dormancy release, thereby avoiding the interference of bud growth with differentially expressed data at three stages of chilling stress to a certain extent. Altogether, we therefore used artificial chilling treatment to maintain an individual and stable cold stress on tree peony buds. We believe that it could be useful to screen differentially expressed genes involved in bud dormancy regulation through RNA-Seq method.

**References:**

**Gao L, Niu D, Chi T, Yuan Y, Liu C, Gai S, Zhang Y** (2023) PsRGL1 negatively regulates chilling-and gibberellin-induced dormancy release by PsF-box1-mediated targeting for proteolytic degradation in tree peony. Hort Res **10:** uhad044

**Huang X, Zhu W, Dai S, Gai S, Zheng G, Zheng C** (2008) The involvement of mitochondrial phosphate transporter in accelerating bud dormancy release during chilling treatment of tree peony (*Paeonia suffruticosa*). Planta **228:** 545-552

**Zhang L, Cheng F, Huang H, Geng Z, He C** (2023) PsFT, PsTFL1, and PsFD are involved in regulating the continuous flowering of tree peony (*Paeonia* × *lemoinei* 'High Noon'). Agronomy **13:** 2071

**Zhang T, Yuan Y, Zhan Y, Cao X, Liu C, Zhang Y, Gai S** (2020). Metabolomics analysis reveals Embden Meyerhof Parnas pathway activation and flavonoids accumulation during dormancy transition in tree peony. BMC Plant Biol **20:** 484

**Zhang YX, Yu D, Tian XL, Liu CY, Gai SP, Zheng GS** (2015) Differential expression proteins associated with bud dormancy release during chilling treatment of tree peony (*P aeonia suffruticosa*). Plant Biol **17:** 114-122

**Comment 3:**

*Figure 1: In A, the image is the pot of a petunia where you can observe the buds. However, in Figure 5, the OE of PsMU306 is shown at 10 days after sowing (DAS). The relative expression levels (B) is indicated in young leaves at 10DAS, however the whole story goes about the bud dormancy. Why these experiments shown are not done in buds but in young*

*leaves?? How are the buds after 10DAS? Could we see a picture of that? The G pictures, are done at 50DAS?, where and how are the buds over there? If the leaves are already formed, there is no dormancy over there. So, the whole point to show the role of MYB306 and the subsequent genes which it interacts in dormancy is not really provided, or am I wrong?*

**Response:**

Thanks for the reviewer's insightful comments. The image in Figure 1A is actually the pot of 5-year-old grafted tree peony plants where the apical buds can be observed. It is absolutely true that the whole story goes about the bud dormancy. To dissect the regulatory mechanisms underlying bud dormancy release, a MYB transcription factor *PsMYB306* was selected from RNA-Seq data for functional characterization in tree peony. However, considering the recalcitrance of tree peony plants to stable genetic transformation (Luan et al., 2023), we used transient TRV-based VIGS method to down-regulate *PsMYB306*. For the overexpression assay, it is quite difficult to build an ideal model platform for the studies of bud dormancy based on the structural specificity of woody perennial tree peony buds. Notably, it has been widely recognized that bud dormancy and seed dormancy exhibit a high similarity in regulatory mechanisms (Chao et al., 2015; Wang et al., 2016), in which the endogenous hormones ABA and GAs also play important roles (Liu et al., 2010; Chen et al., 2020). It would be feasible to analyze the function of *PsMYB306* in bud dormancy through seed dormancy assay in model plants. This notion has been supported by some previous studies on bud dormancy regulation (Li et al., 2021; Fan et al., 2023; Wang et al., 2023). Petunia, as a small flowering herbaceous plant, has been widely employed as a model platform for molecular studies of floral crops (Sun et al., 2019). Accordingly, we chose petunia as a model plant to perform *PsMYB306* overexpression assay.

In Figure 5, no visible buds existed in the newly germinated petunia seedlings at 10 DAS, so we collected young leaves to perform gene expression analysis. No picture can be shown for the buds at 10 DAS. As explained above, the objective of this assay in petunia is to examine the effect of *PsMYB306* overexpression on seed dormancy release and germination. Although a few axillaris buds might exist in the petunia plants at 50 DAS (Figure 5G), which is already at the later stage of plant growth. Especially, there is no dormancy phenomenon over there at 50 DAS. It is very different from the bud dormancy at the earlier stage of tree peony growth. The reason we investigated the petunia plants at 50 DAS is to further determine the influence of *PsMYB306* overexpression on ABA- and GA-mediated plant growth and development. Many pieces of evidence have suggested that GAs promote stem elongation and flowering (Hisamatsu et al., 2000; Shan et al., 2021), whereas ABA has

opposite effects (Garcia-Luis et al., 1986; Jarret and Gawel, 1991). Our results showed that *PsMYB306* overexpression inhibited plant growth and flowering, and increased and decreased ABA and bioactive GA levels, respectively (Figure 5H-K). These data further provided the evidence for the modulation of ABA and GAs by PsMYB306. According to the statement above, we hold an opinion that the overexpression assay in petunia supports the findings and conclusions from the VIGS assay in tree peony, suggesting the negative role of PsMYB306 in bud dormancy release by modulating ABA and GA levels.

**References:**

**Chao WS, Doğramacı M, Horvath DP, Foley ME, Anderson JV** (2015) Dormancy induction and release in buds and seeds. Adv Plant Dorm, 235-256

**Chen H, Ruan J, Chu P, Fu W, Liang Z, Li Y, Tong J, Xiao L, Liu J, Li C, Huang S** (2020). AtPER1 enhances primary seed dormancy and reduces seed germination by suppressing the ABA catabolism and GA biosynthesis in Arabidopsis seeds. Plant J **101:** 310-323

**Fan X, Zou X, Fu L, Yang Y, Li M, Wang C, Sun H** (2023) The RING-H2 gene LdXERICO plays a negative role in dormancy release regulated by low temperature in *Lilium davidii* var. unicolor. Hort Res **10:** uhad030

**Garcia-Luis A, Almela V, Monerri C, Agustí M, Guardiola JL** (1986) Inhibition of flowering in vivo by existing fruits and applied growth regulators in *Citrus unshiu*. Physiol Plantarum **66:** 515-520

**Hisamatsu T, Koshioka M, Kubota S, Fujime Y, King RW, Mander LN** (2000) The role of gibberellin biosynthesis in the control of growth and flowering in *Matthiola incana*. Physiol Plantarum **109:** 97-105

**Jarret RL, Gawel N** (1991) Abscisic acid-induced growth inhibition of sweet potato (*Ipomoea batatas* L.) in vitro. Plant Cell Tiss Org **24:** 13-18

**Li Y, An S, Cheng Q, Zong Y, Chen W, Guo W, Zhang L** (2021) Analysis of evolution, expression and genetic transformation of TCP transcription factors in blueberry reveal that VcTCP18 negatively regulates the release of flower bud dormancy. Front Plant Sci **12:** 697609

**Liu Y, Ye N, Liu R, Chen M, Zhang J** (2010) $H_2O_2$ mediates the regulation of ABA catabolism and GA biosynthesis in Arabidopsis seed dormancy and germination. J Exp Bot **61:** 2979-2990

**Luan Y, Chen Z, Tang Y, Sun J, Meng J, Tao J, Zhao D** (2023) Tree peony PsMYB44

negatively regulates petal blotch distribution by inhibiting dihydroflavonol-4-reductase gene expression. Ann Bot **131:** 323-334

**Shan F, Zhang R, Zhang J, Wang C, Lyu X, Xin T, Yan C, Dong S, Ma C, Gong, Z** (2021) Study on the regulatory effects of $GA_3$ on soybean internode elongation. Plants, **10:** 1737

**Sun D, Zhang X, Zhang Q, Ji X, Jia Y, Wang H, Niu L, Zhang Y** (2019) Comparative transcriptome profiling uncovers a *Lilium regale* NAC transcription factor, *LrNAC35*, contributing to defence response against cucumber mosaic virus and tobacco mosaic virus. Mol Plant Pathol **20:** 1662-1681

**Wang D, Gao Z, Du P, Xiao W, Tan Q, Chen X, Li L, Gao D** (2016) Expression of ABA metabolism-related genes suggests similarities and differences between seed dormancy and bud dormancy of peach (*Prunus persica*). Front Plant Sci **6:** 1248

**Wang L, Sun W, Liu X, Xu Y, Lyu Z, Liu R, Jiu S, Zhang C, Wang J** (2023) Genome-wide identification of the *NCED* gene family and functional characterization of PavNCED5 related to bud dormancy in sweet cherry. Sci Hortic **319:** 112186

**Comment 4:**

*Figure 2: should be explain the genes described, especially when along the text where not explained, like e.g. PYL/PYR/RCAR.*

**Response:**

Thanks for the reviewer’s helpful advice. According to the suggestion, we added some clear explanations for the differentially-expressed genes described in Figure 2 in the section of Results (Line 180-182, 188-190).

**Comment 5:**

*The quality of the figures in the merged PDF is not sufficient for the quality of this journal.*

**Response:**

We are grateful to the reviewer for the helpful comment. There are relatively more data, graphs, or panels shown in some Figures, and the merged PDF might compress the pixels of the images to some extent. Therefore, some Figures could not be clearly seen. To improve the quality, we increased the pixels of the Figures from 200 dpi to 300 dpi in our resubmitted manuscript. Hopefully, our modifications would meet the requirements of this journal for figure quality.

02-Dec-2023

Prof. Daoyang Sun
Northwest A&F University
College of Landscape Architecture and Arts
Yangling, Shaanxi Province 712100
China

MSID: PP2023-RA-01764D
MS TITLE: An abscisic acid-induced transcription factor, PsMYB306, negatively regulates tree peony bud dormancy release.

Dear Dr. Daoyang Sun:

I am pleased to inform you that your revised manuscript "An abscisic acid-induced transcription factor, PsMYB306, negatively regulates tree peony bud dormancy release." has been accepted for publication in Plant Physiology. Your manuscript will be checked for consistency with journal formatting and image standards by one of our Science Editors, who will contact you shortly. They may have a number of queries and suggestions for you. Please keep in mind that they work to increase the online visibility of your article and its attraction for readers from the widest of backgrounds. You will have the opportunity to review any revisions, and I hope you will work constructively with them to get the most out of your research. You will also have the opportunity to review all changes when you receive the author proofs for your article.

Once validated for publication, your article will be sent to a Science Editor for a final review of your manuscript and source files. After your final files are uploaded, your article will appear in the next available issue and the online Preview version of your article will be posted after your signed Copyright and Checklist forms and final source files are received.

Please be sure to include the following in your manuscript file:
Harvard style citations
List of all author contributions and funding information at the beginning of manuscript after the titles and author list.
List all supplemental materials associated with the article, with titles, at the end of the ms, before the references and acknowledgments section.
Complete Figure Legends for all figures appearing in the article listed before the Literature Cited.

Thank you and congratulations on your Plant Physiology paper!

Sincerely,
Board of Editors, Plant Physiology

=====
FIRST AUTHOR PROFILES
=====

We recognize the hard work that goes into being First Author of a Plant Physiology paper by publishing a "First Author Feature" for each First Author alongside your paper.

If the first author(s) would like to submit information for a feature on Facebook and Twitter, fill out the form by clicking on the link below:

Plant Physiology First Author Feature Form: https://forms.office.com/r/ZBCGZfhm8F
If you cannot access the form through the link above, please contact Rachel at rbelsky@aspb.org.

To upload an image of yourself to accompany your profile, please put your photo in the correct journal folder at the link below. You must label your image: Author First Name Last Name Article Title.

First Author Profile Image Dropbox: https://www.dropbox.com/scl/fo/xz029p7qjr2jml2rp2kmq/h?dl=0&rlkey=6fkxcaqmxx35ap152b2osy1fz

=====

IMPORTANT REMINDER: PEER REVIEW REPORTS
=====

If you opted to publish a peer review report along with your article during the original submission process, it will be prepared by the editorial staff and publicly posted with your manuscript, inside the zip file that contains any other supplemental material. As a reminder, the peer review report is a public record of all comments from editors and reviewers, as well as your prior responses, as you received them in the decision letters for each draft of your manuscript. If you agreed to publish this report and have changed your mind, or are not sure if you selected this option, please contact the editorial office as soon as possible before signing the license agreement from our publisher.

---------------------------------------------------------------------------- Reviewer comments:
Reviewer #1 (Comments for the Author):

Thank you for a very careful and thoughtful revision. I still am not convinced that the work is novel as the relationship between MYB306, ABA production and subsequent impact on GA levels and the well characterized role of GAs in regulating ecodormancy release has been established in model systems. However, the science is sound and the results are interesting and convincing in so much as it demonstrates that this regulatory module is functional in tree peony as well.

Reviewer #2 (Comments for the Author):

This re-submission is a better version of the previous one. The authors have changed and make corrections when required. The number of repetitions are sufficient for this type of experiment. Therefore, I would recommend it in its current form.
